# Assessing the reporting quality of pediatric neuro-oncology protocols, abstracts, and trials: Adherence to the SPIRIT and CONSORT statements

**DOI:** 10.1093/nop/npae042

**Published:** 2024-05-11

**Authors:** Joshua S Suppree, Sophia Hart, Sumirat M Keshwara, Sandhya Trichinopoly Krishna, Conor S Gillespie, George E Richardson, Mohammad A Mustafa, Conor L Mallucci, Barry Pizer, James Hayden, Abdurrahman I Islim, Michael D Jenkinson, Christopher P Millward

**Affiliations:** School of Medicine, University of Liverpool, Liverpool, UK; Department of Neurosurgery, The Walton Centre NHS Foundation Trust, Liverpool, UK; Department of Neurosurgery, The Walton Centre NHS Foundation Trust, Liverpool, UK; Department of Neurosurgery, The Walton Centre NHS Foundation Trust, Liverpool, UK; Department of Neurosurgery, The Walton Centre NHS Foundation Trust, Liverpool, UK; School of Medicine, University of Liverpool, Liverpool, UK; Department of Neurosurgery, The Walton Centre NHS Foundation Trust, Liverpool, UK; School of Medicine, University of Liverpool, Liverpool, UK; Department of Neurosurgery, The Walton Centre NHS Foundation Trust, Liverpool, UK; Department of Neurosurgery, The Walton Centre NHS Foundation Trust, Liverpool, UK; Department of Pediatric Neurosurgery, Alder Hey Children’s NHS Foundation Trust, Liverpool, UK; University of Liverpool, Liverpool, UK; Department of Pediatric Oncology, Alder Hey Children’s NHS Foundation Trust, Liverpool, UK; Department of Pediatric Oncology, Alder Hey Children’s NHS Foundation Trust, Liverpool, UK; Department of Pharmacology & Therapeutics, Institute of Systems, Molecular, & Integrative Biology, University of Liverpool, Liverpool, UK; Department of Neurosurgery, The Walton Centre NHS Foundation Trust, Liverpool, UK; Department of Pediatric Oncology, Alder Hey Children’s NHS Foundation Trust, Liverpool, UK; Department of Pharmacology & Therapeutics, Institute of Systems, Molecular, & Integrative Biology, University of Liverpool, Liverpool, UK; Department of Neurosurgery, The Walton Centre NHS Foundation Trust, Liverpool, UK; Department of Pediatric Oncology, Alder Hey Children’s NHS Foundation Trust, Liverpool, UK; Department of Pharmacology & Therapeutics, Institute of Systems, Molecular, & Integrative Biology, University of Liverpool, Liverpool, UK; Department of Neurosurgery, The Walton Centre NHS Foundation Trust, Liverpool, UK

**Keywords:** clinical trial, CONSORT, CONSORT-A, SPIRIT

## Abstract

**Background:**

It is of vital importance to comprehensively and transparently report clinical trial activity. The SPIRIT 2013 and CONSORT 2010 statements exist to define items to be reported in clinical trial protocols and randomized controlled trials, respectively. The aim of this methodological review was to assess the reporting quality of pediatric neuro-oncology trial protocols and trial result articles.

**Methods:**

Published trial protocols and phase II/III trial result articles relating to pediatric brain tumors (published after the introduction of the SPIRIT 2013 statement), were identified through searches of 4 electronic bibliographic databases. The reporting quality of included trial protocols and result articles was assessed against the aforementioned statements. In addition, the CONSORT-A checklist was used to assess the abstracts of trial result articles. Percentage adherence was calculated for each article.

**Results:**

Nine trial protocols, 68 phase II trials, and 8 phase III trial result articles were included. Mean adherence of trial protocols to the SPIRIT statement was 76.8% (*SD*: 0.09). Mean adherence of trial abstracts to CONSORT-A was 67.4% (*SD*: 0.13) for phase II abstracts and 47.5% (*SD*: 0.09) for phase III abstracts. Adherence of trial result articles to CONSORT was 71.3% (*SD*: 0.10) for phase II trials and 70.3% (*SD*: 0.13) for phase III trials.

**Conclusions:**

The reporting quality of pediatric neuro-oncology trial protocols and trial result articles requires improvement, particularly in the areas of randomization and blinding. This is consistent with our previously published findings following similar assessment of reporting quality for adult neuro-oncology trial protocols and result articles.

Pediatric brain tumors are the most common solid tumor in children, are associated with a poor prognosis, and are the leading cause of cancer-related deaths in this population.^1^ Clinical trials for pediatric brain tumors are essential in order to advance treatment options and refine treatment algorithms. Comprehensive and transparent presentation of both the intended trial (trial protocol) and the trial results (trial results article), regardless of the actual trial result, is an essential requirement to facilitate knowledge communication and advancement. A uniform approach to reporting essential trial details can be achieved by uniformly adhering to published statements that offer a checklist of key items to be included.

The SPIRIT (Standard Protocol Items: Recommendations for Interventional Trials) 2013 statement serves as a vital resource, offering evidence-based guidelines for crafting comprehensive clinical trial protocols.^2^ Endorsed by a wide array of stakeholders including journals, regulators, and academic research institutions, the SPIRIT statement provides a robust framework consisting of 51 items seen as vital for protocol reporting.^2,3^ By adhering to the SPIRIT recommendations, researchers ensure the proactive integration of critical methodological facets essential to the robust design of a clinical trial. This strategic adherence not only elevates the quality of trial planning but also proactively addresses potential pitfalls and biases, thereby enhancing the credibility and dependability of the trial outcomes.

The CONSORT (Consolidated Standards of Reporting Trials) statement, plays a pivotal role in providing evidence-based recommendations aimed at elevating the quality of reporting for Randomized Controlled Trials (RCTs). This influential framework, last updated in 2010, has garnered the support of a vast network of over 600 medical journals, underlining its accepted role for the presentation of clinical trial results.^4,5^ Comprising 25 distinct items, the CONSORT statement considers the reporting of clinical trial conception, execution, analysis, and interpretation. By emphasizing the incorporation of these comprehensive guidelines, the overarching objective centers on promoting consistency in the way trials are reported, ensuring that key details are effectively communicated to researchers, clinicians, and the broader scientific community. The CONSORT framework has evolved to encompass specialized extensions like CONSORT-A. This specific extension addresses the requirements for reporting informative clinical trial abstracts.^6^

When considering both the SPIRIT and CONSORT statements collectively, they provide universally accepted directives for reporting, serving as invaluable tools for individuals aiming to proficiently convey their intended and executed randomized controlled trials. Deviating from these established standards in either the protocol or results article might impede the trial’s potential to contribute to informed clinical decision-making.

A review assessing the reporting quality of adult neuro-oncology protocols, trials, and abstracts has been published.^7^ The review identified 7 trial protocols and 36 clinical trial result articles. The average conformity of trial protocols with the SPIRIT statement was 79.4% (*SD*: 0.11). The average adherence of clinical trial abstracts to CONSORT-A was 75.3% (*SD*: 0.12), whilst the average adherence to CONSORT was 74.5% (*SD*: 0.10). It was concluded that improvement was needed to ensure the transparent communication of clinical trials, and their results, with the literature. The standard of reporting quality in pediatric neuro-oncology trial protocols and clinical trial result articles has not been assessed to date. The aim of this methodological review was to assess the reporting quality of pediatric phase II and phase III neuro-oncology trial result articles and published trial protocols published from 2014 onwards against the SPIRIT and CONSORT statements. This review is the second and final methodological review addressing the reporting quality of neuro-oncology protocols, trials and trial abstracts, and in doing so, we intend for reporting standards in neuro-oncology to improve.

## Material and Methods

The methodology utilized in this study closely parallels that of our previously published paper.^7^ Given the substantial alignment between the 2 research papers, the methodological descriptions within this paper have been abbreviated.

### Information Sources

Electronic bibliographic databases including PubMed, EMBASE, CINAHL, and the Cochrane central register of controlled trials were searched to identify protocols, results articles, and abstracts of pediatric phase II and phase III neuro-oncology studies published since 2014. The complete search strategies are provided in Supplementary Appendix 1.

### Eligibility Criteria

Pediatric phase II and phase III neuro-oncology protocols, result articles, and abstracts were evaluated using the SPIRIT, CONSORT, and CONSORT-A statements. Protocols, result articles, and abstracts that belonged to the same study were evaluated using the corresponding statements in the same way as stand-alone publications. The inclusion criteria for eligible studies was specified as published clinical trial protocols and clinical trial result papers that describe cohorts of children and young adults (minimum 10 patients) with an intracranial tumor receiving interventions including perioperative care, surgery, radiotherapy, pharmacotherapy, or any combination of the above. The full eligibility criteria are provided in Supplementary Appendix 2.

### Study Selection and Data Extraction

Searches were downloaded from their online databases for the purpose of deduplication and screening. Screening was performed by 2 review authors (J.S. and S.H.) and any titles that did not achieve concordance were highlighted within the platform, discussed, and resolved between the 2 review authors in person or escalated to another review author (S.T.K.). The review was reported in accordance with the Preferred Reporting Items for Systematic Reviews and Meta-analysis (PRISMA) guidelines, where applicable.^8^

### Assessment of Reporting Quality

The SPIRIT 2013 statement, comprising 51 items (Supplementary Appendix Table 3), was used to assess the reporting quality of trial protocols. Each item was assigned a point based on adequate reporting in the manuscript or Supplementary Material (Yes = 1 point, No = 0 points). Nonapplicable items reduced the maximum attainable score by 1 point (for instance, criterion not applicable to phase II clinical trials). The maximum score applicable to all eligible protocols was 51. The CONSORT-A and CONSORT checklists were used to evaluate clinical trial abstracts and result articles.

When assessing the reporting quality of phase II abstracts and trials, a modified CONSORT-A (Supplementary Appendix 4) and CONSORT 2010 (Supplementary Appendix 6) checklist was used. Checklist items 8 and 9 on the CONSORT-A checklist relate to randomization and were ignored in such circumstances, in order to not adversely affect the overall reporting quality. Moreover, when assessing phase II trials using the CONSORT 2010 statement question 1a “Identification of the study as randomized” was altered to identifying the study as a phase II trial to avoid penalizing trails that were not randomized. Similarly, items on the CONSORT 2010 checklist relating to randomization (question 8a to question 10) and blinding (questions 11a to 11b) were not applicable to the trials as they were not randomized. Instead of receiving a “No” for these questions, a “N/A” was given and the overall percentage was modified when statistical analysis was carried out. If a phase II trial was randomized and failed to report information on the checklist items they would receive a “No” for this item.

### Statistical Analysis

Descriptive analysis was used to calculate the proportion (shown as a percentage) of SPIRIT statement items that were adequately reported in protocols. Mean values are presented alongside the standard deviation. The same analysis was carried out on the clinical trial abstracts and result articles. Additional analysis based on the year of publication was also carried out to explore possible relationships between the time elapsed since the publication of the guideline and the level of adherence reported.

## Results

### Study Characteristics

**Figure 1.**
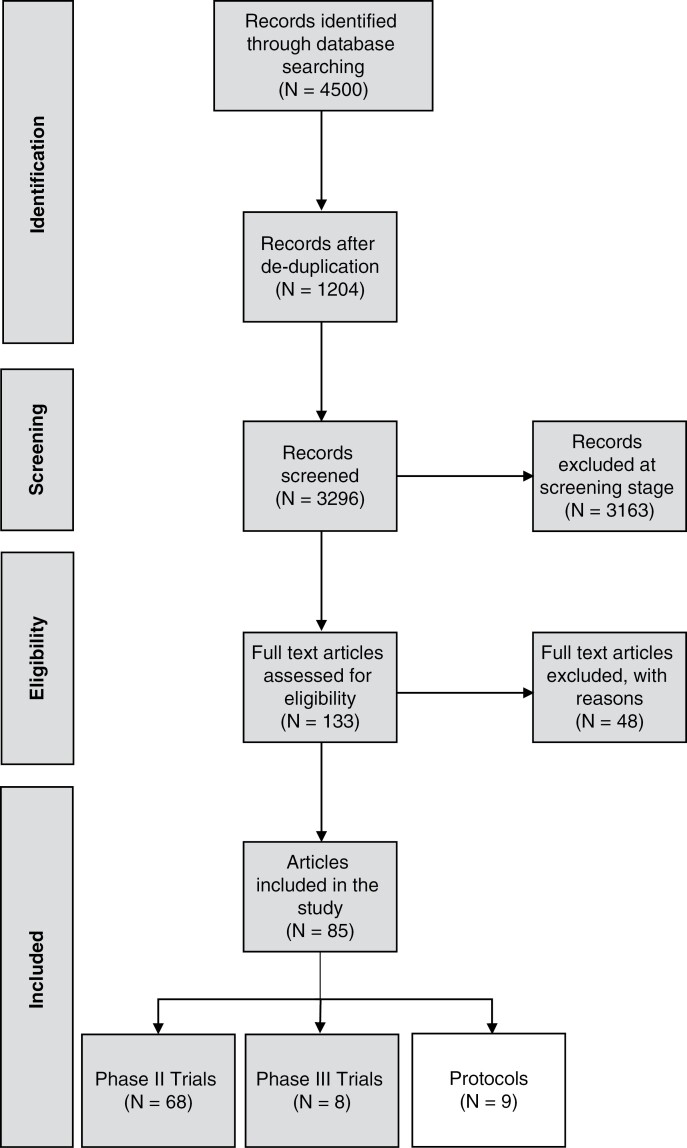
PRISMA flow chart.

Eighty-five articles were included in this review—9 trial protocols, 68 phase II clinical trials, and 8 phase III clinical trials. The search, screening, and selection of results are summarized in Figure 1. Of the included protocols 6 (66.7%) had a first author affiliated with an institution in Europe. Medulloblastoma was the most common tumor investigated in the protocols accounting for 66.7% (*n* = 6). Table 1 provides an overview of the included protocols.

**Table 1.** Overview of Included Protocols

	1st Author	Country	Year	Trial name	Tumor type
1	Frank Deinlein	Europe	2006	radiation therapy and combination chemotherapy in treating young patients with medulloblastoma, supratentorial primitive neuroectodermal tumor, or ependymoma	Medulloblastoma
2	James M Olson	USA	2006	chemotherapy and radiation therapy in treating young patients with newly diagnosed, previously untreated, high-risk medulloblastoma	Medulloblastoma
3	Stefan Rutkowski	Europe	2006	Radiation therapy and combination chemotherapy in treating young patients with medulloblastoma, supratentorial primitive neuroectodermal tumor, or ependymoma	Medulloblastoma
4	Jeff M. Michalski	Europe	2009	Children’s oncology group phase iii trial of reduced-dose and reduced-volume radiotherapy with chemotherapy for newly diagnosed average-risk medulloblastoma	Medulloblastoma
5	Carin Damen-Korbijn	USA	2012	A study of carboplatin with radiotherapy and isotretinoin in patients with other than average risk medulloblastoma/PNET	Medulloblastoma
6	Amy A Smith	USA	2013	ACNS0831: a children’s oncology group phase III randomized trial of post-radiation chemotherapy in patients with newly diagnosed ependymoma ages 1–21 years	Ependymoma
7	MC Le Deley	Europe	2013	Biomede trial: a randomized trial from the innovative therapies for children with cancer (ITCC) consortium to evaluate new drugs in diffuse intrinsic pontine glioma (DIPG)	Diffuse Intrinsic Pontine Glioma (DIPG)
8	Katja von Hoff	Europe	2015	Study for children with medulloblastoma of standard risk, between 3 and 5 years old and <22 years old	Medulloblastoma
9	Pierre Leblond	Europe	2016	SIOP Ependymoma II—an International Clinical Program for the diagnosis and treatment of children, adolescents, and young adults with ependymoma	Ependymoma

Fifty-three percent (*n* = 19) of the phase II clinical trial articles had a first author affiliated with an institution in the United States, whilst 25% (*n* = 9) were affiliated with an institution in Europe, and the remainder from the rest of the world (22% (*n* = 8)). High grade glioma was the study subject for 20.6% (*n* = 14) and diffuse intrinsic pontine glioma was the subject of 16.2% (*n* = 11) phase II trials. Table 2 provides an overview of the included clinical trial articles.

**Table 2.** Overview of Included Trials and Abstracts

	1st Author	Country	Year	Journal	Trial name	Trial phase	Tumor type
1	Sridharan Gururangan	USA	2002	Journal of Clinical Oncology	Carboplatin in children with progressive low-grade gliomas	II	LGG
2	Mark T Jennings	USA	2002	Journal of Clinical Oncology	Preradiation chemotherapy in primary high-risk brainstem tumors	II	Brainstem gliomas
3	Stewart J Kellie	Australia	2002	Medical and Pediatric Oncology	Activity of postoperative carboplatin, etoposide, and high-dose methotrexate in pediatric CNS embryonal tumors	II	CNS embyronal tumors
4	L S Lashford	UK	2002	Journal of Clinical Oncology	Temozolomide in malignant gliomas of childhood	II	Malignant glioma
5	Thomas E Merchant	USA	2002	International Journal of Radiation Oncology Biology Physics	Conformal radiation therapy for pediatric patients with localized low-grade astrocytoma and ependymoma	II	Low-grade astrocytoma and ependymoma
6	Thomas E Merchant	USA	2004	Journal of Clinical Oncology	Conformal radiation therapy and evaluation of radiation-related CNS effects for pediatric patients with localized ependymoma	II	Localized ependymoma
7	Stanislaw R Burzynski	USA	2005	Integrative Cancer Therapies	Long-term survival of high-risk pediatric patients with primitive neuroectodermal tumors treated with antineoplastons A10 and AS2-1	II	Primitive neuroectodermal tumors
8	Tobey J MacDonald	USA	2005	Cancer	High-dose chemotherapy before radiation in children with newly diagnosed high-grade astrocytoma	II	High-grade astrocytoma
9	Murali M Chintagumpala	USA	2006	Journal of Neuro-Oncology	Procarbazine and topotecan in children with high-grade glioma	II	HGG
10	Maryam Fouladi	USA	2006	Cancer	Oxaliplatin in children with recurrent or refractory medulloblastoma, supratentorial primitive neuroectodermal tumors, and atypical teratoid rhabdoid tumors	II	Recurrent or refractory medulloblastoma, supratentorial primitive Neuroectodermal tumors, and atypical teratoid rhabdoid tumors
11	Katherine Warren	USA	2006	Cancer Chemotherapy and Pharmacology	Intravenous lobradimil and carboplatin in childhood brain tumors	II	Brainstem glioma, high-grade glioma, low-grade glioma, 1medullobastoma/primitive neuroectodermal tumor (PNET), and ependymoma
12	Maryam Fouladi	USA	2007	Cancer	Farnesyl transferase inhibitor, tipifarnib, in children with recurrent or progressive high-grade glioma, medulloblastoma/primitive neuroectodermal tumor, or brainstem glioma	II	recurrent or progressive high-grade glioma, medulloblastoma/primitive neuroectodermal tumor, or brainstem glioma
13	H Stacy Nicholson	USA	2007	Cancer	Temozolomide in children and adolescents with recurrent central nervous system tumors	II	CNS tumors—mainly high/low grade astrocytoma, medulloblastoma, and PNET
14	David N Korones	USA	2008	Pediatric Blood & Cancer	Treatment of children with diffuse intrinsic brain stem glioma with radiotherapy, vincristine, and oral VP-16	II	DIPG
15	Jeffrey Allen	USA	2009	International Journal of Radiation Oncology Biology Physics	Preradiotherapy chemotherapy followed by hyperfractionated radiotherapy for newly diagnosed high-risk medulloblastoma/primitive neuroectodermal tumor	II	Medulloblastoma/primitive neuroectodermal tumor
16	Sylvain Baruchel	Canada	2009	European Journal of Cancer	Imatinib in recurrent and refractory pediatric central nervous system tumors	II	CNS tumors—mainly astrocytoma, ependymoma, GBM, medulloblastoma
17	Sridharan Gururangan	USA	2010	Journal of Clinical Oncology	Lack of efficacy of bevacizumab plus irinotecan in children with recurrent malignant glioma and diffuse brainstem glioma	II	Recurrent malignant glioma and diffuse brainstem glioma
18	Daphne A. Haas-Kogan	USA	2011	Neuro-oncology	Tipifarnib and radiation in children with newly diagnosed diffuse intrinsic pontine gliomas	II	DIPG
19	Jane E Minturn	USA	2011	Pediatric Blood & Cancer	Metronomic oral topotecan for recurrent childhood brain tumors	II	CNS tumors—mainly ependymoma, high-grade glioma (HGG), brainstem glioma, and primitive neuroectodermal tumor
20	Ian F Pollack	USA	2011	Neuro-oncology	Gefitinib and irradiation in children with newly diagnosed brainstem gliomas	II	Diffuse intrinsic brainstem glioma.
21	James H Garvin Jr	USA	2012	Pediatric Blood & Cancer	Pre-irradiation chemotherapy for childhood intracranial ependymoma	II	Intracranial ependymoma
22	Birgit Geoerger	France	2012	European Journal of Cancer	Temsirolimus in children with high-grade glioma, neuroblastoma and rhabdomyosarcoma	II	High-grade glioma, neuroblastoma and rhabdomyosarcoma
23	Katherine Warren	USA	2012	Cancer	Study of pegylated interferon α-2b (PEG-Intron(®)) in children with diffuse intrinsic pontine glioma	II	Diffuse intrinsic pontine glioma
24	Katherine Warren	USA	2012	Journal of Neuro-Oncology	Study of O6-benzylguanine and temozolomide in pediatric patients with recurrent or progressive high-grade gliomas and brainstem gliomas	II	High-grade gliomas and brainstem gliomas
25	Kristin A Bradley	USA	2013	International Journal of Radiation Oncology Biology Physics	Motexafin-gadolinium and involved field radiation therapy for intrinsic pontine glioma of childhood	II	Intrinsic pontine glioma
26	Jacques Grill	France	2013	Neuro-oncology	Irinotecan in combination with temozolomide (TEMIRI) in children with recurrent or refractory medulloblastoma	II	Medulloblastoma
27	Darren Hargrave	UK	2013	Journal of Neuro-Oncology	Irinotecan in combination with temozolomide (TEMIRI) in children with newly diagnosed high-grade glioma	II	HGG
28	Tobey J MacDonald	USA	2013	Neuro-oncology	Cilengitide in the treatment of refractory or relapsed high-grade gliomas in children	II	HGG
29	Anne B Warwick	USA	2013	Pediatric Blood & Cancer	Pemetrexed in children and adolescents with refractory solid tumors	II	Refractory solid tumors including ependymoma, Ewing sarcoma, medulloblastoma, neuroblastoma, osteosarcoma
30	Angela Di Giannatale	France	2014	European Journal of Cancer	Temozolomide in combination with topotecan (TOTEM) in relapsed or refractory neuroblastoma	II	Relapsed or refractory neuroblastoma
31	Matthias A Karajannis	USA	2014	Neuro-oncology	Sorafenib in children with recurrent or progressive low-grade astrocytomas	II	Recurrent or progressive low-grade astrocytomas
32	C. Kretschmar	USA	2014	Pediatric Blood & Cancer	Pre-radiation chemotherapy with response-based Radiation therapy in children with central nervous system germ cell tumors	II	Germ cell tumors—mainly germinoma
33	Maryam Fouladi	USA	2014	Journal of Neuro-Oncology	A molecular biology and phase II trial of lapatinib in children with refractory CNS malignancies: a pediatric brain tumor consortium study	II	CNS tumors—medulloblastoma, HGG, ependymoma
34	Ute Bartels	Canada	2014	Neuro-oncology	Phase 2 study of safety and efficacy of nimotuzumab in pediatric patients with progressive diffuse intrinsic pontine glioma	II	DIPG
35	Stanislaw R Burzynski	USA	2014	Child’s Nervous System	The response and survival of children with recurrent diffuse intrinsic pontine glioma based on phase II study of antineoplastons A10 and AS2-1 in patients with brainstem glioma	II	Recurrent pediatric diffuse intrinsic pontine gliomas (RPDIPG)
36	Graziella Cefalo	Italy	2014	Neuro-oncology	Temozolomide is an active agent in children with recurrent medulloblastoma/primitive neuroectodermal tumor: an Italian multi-institutional phase II trial	II	Recurrent medulloblastoma/primitive neuroectodermal tumor
37	Maura Massimino	Italy	2014	Journal of Neuro-Oncology	Results of nimotuzumab and vinorelbine, radiation and re-irradiation for diffuse pontine glioma in childhood	II	DIPG
38	Tamara Vern-Gross	USA	2014	Neuro-Oncology	Prospective evaluation of local control and late effects of conformal radiation therapy in children, adolescents, and young adults with high-grade glioma	II	HGG
39	Nathan J Robison	USA	2014	Pediatric Blood & Cancer	A phase II trial of a multi-agent oral antiangiogenic (metronomic) regimen in children with recurrent or progressive cancer	II	HGG, ependymoma, LGG, medulloblastoma/PNET, neuroblastoma
40	Patricia Robinson	USA	2014	Journal of Neuro-Oncology	Multimodality therapy for CNS mixed malignant germ cell tumors (MMGCT): Results of a phase II multi-institutional study	II	CNS mixed malignant germ cell tumors (MMGCT)
41	Ibrahim Qaddoumi	USA	2014	Frontiers in Oncology	Phase II trial of erlotinib during and after radiotherapy in children with newly diagnosed high-grade gliomas	II	HGG (AA and GBM)
42	Diana Osorio	USA	2014	Journal of Neuro-Oncology	Pre-irradiation intensive induction and marrow-ablative consolidation chemotherapy in young children with newly diagnosed high-grade brainstem gliomas: report of the “head-start” I and II clinical trials	II	High-grade brainstem glioma
43	Orren Beaty	USA	2015	Pediatric Blood & Cancer	A Phase II trial and pharmacokinetic study of oxaliplatin in children with refractory solid tumors: a children’s oncology group study	II	Ewing sarcoma/peripheral PNET, osteosarcoma, rhabdomyosarcoma, neuroblastoma, high- and low-grade astrocytoma, brain stem glioma, ependymoma, hepatoblastoma and selected rare tumors
44	Bruce Cohen	USA	2015	Pediatric Neurology	Pilot study of intensive chemotherapy with peripheral hematopoietic cell support for children <3 years of age with malignant brain tumors, the ccg-99703 phase I/II study. a report from the children’s oncology group	II	CNS tumors—medulloblastoma, supratentorial PNET, ependymoma
45	Mariko DeWire	USA	2015	Journal of Neuro-Oncology	An open-label, 2-stage, phase II study of bevacizumab and lapatinib in children with recurrent or refractory ependymoma: a collaborative ependymoma research network study (CERN)	II	Recurrent or refractory ependymoma
46	Stewart Goldman	USA	2015	Journal of Clinical Oncology	Phase II trial assessing the ability of neoadjuvant chemotherapy with or without second-look surgery to eliminate measurable disease for nongerminomatous germ cell tumors: a children’s oncology group study	II	Nongerminomatous GCTs
47	Lisa L R Hartman	USA	2015	Journal of Pediatric Hematology/Oncology	Pediatric phase II trials of poly-ICLC in the management of newly diagnosed and recurrent brain tumors	II	LGG
48	Regina Jakacki	USA	2016	Neuro-oncology	Phase 2 study of concurrent radiotherapy and temozolomide followed by temozolomide and lomustine in the treatment of children with high-grade glioma: a report of the Children’s Oncology Group ACNS0423 study	II	HGG
49	Regina Jakacki	USA	2016	Journal of Neuro-Oncology	Single-agent erlotinib versus oral etoposide in patients with recurrent or refractory pediatric ependymoma: a randomized open-label study	II	Ependymoma
50	Alvaro Lassaletta	Canada	2016	Journal of Clinical Oncology	Phase II weekly vinblastine for chemotherapy-naïve children with progressive low-grade glioma: A Canadian pediatric brain tumor consortium study	II	LGG
51	Torunn Yock	USA	2016	The Lancet Oncology	Long-term toxic effects of proton radiotherapy for pediatric medulloblastoma: A phase 2 single-arm study	II	Medulloblastoma
52	Cynthia Wetmore	USA	2016	Cancer Medicine	Phase II evaluation of sunitinib in the treatment of recurrent or refractory high-grade glioma or ependymoma in children: a children’s Oncology Group Study ACNS1021	II	HGG and ependymoma
53	Ralph Salloum	USA	2016	Journal of Neuro-Oncology	A molecular biology and phase II study of imetelstat (GRN163L) in children with recurrent or refractory central nervous system malignancies: a pediatric brain tumor consortium study	II	Medulloblastoma, high-grade glioma (HGG) and ependymoma
54	John Lucas Jr	USA	2017	International Journal of Radiation Oncology Biology Physics	Prognostic relevance of treatment failure patterns in pediatric high-grade glioma: Is there a role for a revised failure classification system?	II	HGG
55	Margaret Macy	USA	2017	Pediatric Blood & Cancer	A pediatric trial of radiation/cetuximab followed by irinotecan/cetuximab in newly diagnosed diffuse pontine gliomas and high-grade astrocytomas: A Pediatric Oncology Experimental Therapeutics Investigators’ Consortium study	II	DIPG and high-grade astrocytoma
56	Jacques Grill	France	2018	Journal of Clinical Oncology	Phase II, open-label, randomized, multicenter trial (HERBY) of bevacizumab in pediatric patients with newly diagnosed high-grade glioma	II	HGG
57	Peter Manley	USA	2018	Pediatric Blood & Cancer	A phase 1/2 dose-finding, safety, and activity study of cabazitaxel in pediatric patients with refractory solid tumors including tumors of the central nervous system	II	Recurrent HGG and DIPG
58	Yousra Izzuddeen	India	2019	Journal of Neuro-Oncology	Hypofractionated radiotherapy with temozolomide in diffuse intrinsic pontine gliomas: a randomized controlled trial	II	DIPG
59	Joel Cherlow	USA	2019	International Journal of Radiation Oncology Biology Physics	Conformal radiation therapy for pediatric patients with low-grade glioma: results from the children’s oncology group phase 2 study ACNS0221	II	LGG
60	Jason Fangusaro	USA	2019	The Lancet Oncology	Selumetinib in pediatric patients with BRAF-aberrant or neurofibromatosis type 1-associated recurrent, refractory, or progressive low-grade glioma: a multicentre, phase 2 trial	II	BRAF-aberrant or neurofibromatosis type 1-associated recurrent, refractory, or progressive LGG
61	Jason Fangusaro	USA	2019	Journal of Clinical Oncology	Phase II trial of response-based radiation therapy for patients with localized CNS nongerminomatous germ cell tumors: a children’s oncology group study	II	CNS NGGCTs
62	Darren Hargrave	USA	2019	Clinical Cancer Research	Efficacy and safety of dabrafenib in pediatric patients with BRAF V600 mutation–positive relapsed or refractory low-grade glioma: results from a phase I/IIa study	II	LGG
63	Patricia Baxter	USA	2020	Neuro-oncology	A phase I/II study of veliparib (ABT-888) with radiation and temozolomide in newly diagnosed diffuse pontine glioma: A Pediatric Brain Tumor Consortium study	II	DIPG
64	Stewart Goldman	USA	2020	Neuro-oncology	Phase II study of peginterferon alpha-2b for patients with unresectable or recurrent craniopharyngiomas: a Pediatric Brain Tumor Consortium report	II	Craniopharyngioma
65	Lucie Lafay-Cousin	Canada	2020	Journal of Clinical Oncology	Phase II study of nonmetastatic desmoplastic medulloblastoma in children younger than 4 years of age: a Report of the Children’s Oncology Group (ACNS1221)	II	Nodular desmoplastic medulloblastoma (ND) and medulloblastoma with extensive nodularity (MBEN)
66	Gwénaël Le Teuff	France	2020	Pediatric Blood & Cancer	Phase II study of temozolomide and topotecan (TOTEM) in children with relapsed or refractory extracranial and central nervous system tumors including medulloblastoma with post hoc Ba1ian analysis: A European ITCC study	II	CNS tumors—medulloblastoma and PNET expansion
67	Jack Meng-Fen Su	USA	2020	Pediatric Blood & Cancer	A phase 2 study of valproic acid and radiation, followed by maintenance valproic acid and bevacizumab in children with newly diagnosed diffuse intrinsic pontine glioma or high-grade glioma	II	DIPG and HGG
68	Keiko Okada	Japan	2020	Pediatric Blood & Cancer	Phase II study of reduced-dose craniospinal irradiation and combination chemotherapy for children with newly diagnosed medulloblastoma: a report from Japanese Pediatric brain tumor consortium	II	Medulloblastoma
1	Roger Packer	USA	2006	Journal of Clinical Oncology	Phase III study of craniospinal radiation therapy followed by adjuvant chemotherapy for newly diagnosed average-risk medulloblastoma	III	Average-risk medulloblastoma
2	Birgitta Lannering	Europe	2012	Journal of Clinical Oncology	Hyperfractionated vs conventional radiotherapy followed by chemotherapy in standard-risk medulloblastoma: Results from the randomized multicentre HIT-SIOP PNET 4 Trial	III	Medulloblastoma
3	Nancy Tarbell	USA	2013	Journal of Clinical Oncology	High-risk medulloblastoma: a pediatric oncology group randomized trial of chemotherapy before or after radiation therapy (POG 9031)	III	High-risk medulloblastoma
4	Douglas Strother	USA	2013	Neuro-oncology	Benefit from prolonged dose-intensive chemotherapy for infants with malignant brain tumors is restricted to patients with ependymoma: a report of the Pediatric Oncology Group randomized controlled trial 9233/34	III	Medulloblastoma, ependymoma, supratentorial primitive neuroectodermal tumor (sPNET) and other malignant brain tumors
5	Mohamed S. Zaghloul	Egypt	2014	Radiotherapy & Oncology	Hypofractionated conformal radiotherapy for pediatric diffuse intrinsic pontine glioma (DIPG): A randomized controlled trial	III	DIPG
6	V Batra	USA	2015	Pediatric Blood & Cancer	Long-term survival of children <6 years of age enrolled on the CCG-945 phase III trial for newly-diagnosed high-grade glioma	III	HGG
7	Rakesh Jalali	India	2017	JAMA Oncology	Efficacy of stereotactic conformal radiotherapy vs conventional radiotherapy on benign and low-grade brain tumors: a randomized clinical trial	III	Benign and low-grade tumors
8	Maria Eveslage	Germany	2019	Deutsches Arzteblatt International	The postoperative quality of life in children and adolescents with craniopharyngioma	III	Craniopharyngioma

Fifty percent of the phase III trials had a first author affiliated with an institution in the United States (*n* = 4), whilst only 25% (*n* = 4) had a first author affiliated in Europe. The Journal of Clinical Oncology was the main journal where the phase III trials were published (37.5%, *n* = 3). Pediatric Blood and Cancer, JAMA Oncology, Neuro-oncology, Radiotherapy and Oncology, and Deutsches Arzteblatt International each had 1 phase III trial published respectively (12.5%, *n* = 1). Medulloblastoma was the most common study subject in phase III trials (50%, *n* = 4).

### Quality of Reporting of Clinical Trial Protocols as per SPIRIT 2013 Statement

Nine protocols were included in this review and assessed against the SPIRIT statement. A mean adherence rate of 76.8% (*SD*: 0.09) was observed. The range of compliance with the 51 items in the checklist was 34/51 to 49/51. There was 1 “nonapplicable” question in the SPIRIT statement, relevant to 44.5% of the included protocols (*n* = 4), regarding item 17b “blinding.” If a protocol was not blinded and clearly stated this in the abstract, then it would not be applicable for that protocol to explain the methods used to carry out blinding. All included protocols reported the administrative information for the protocol, described the background and rationale, and reported the study setting. Only 77.8% (*n* = 7) of the protocols explained the role of the study sponsor and funders.

Although all 7 protocols described the planned interventions in each group, including administration methods, only 66.7% (*n* = 6) of protocols described criteria for discontinuing or modifying the allocated interventions, as well as strategies to improve protocol adherence. Furthermore only 55.5% (*n* = 5) of protocols listed concomitant care and interventions that would be allowed or prohibited throughout the trial. The assignment of interventions, including details on sequence generation, was reported in 55.5% (*n* = 5) of protocols and allocation and implementation in only 22.2% (*n* = 2). Only 44.4% (*n* = 4) of protocols reported whether blinding took place.

**Figure 2.**
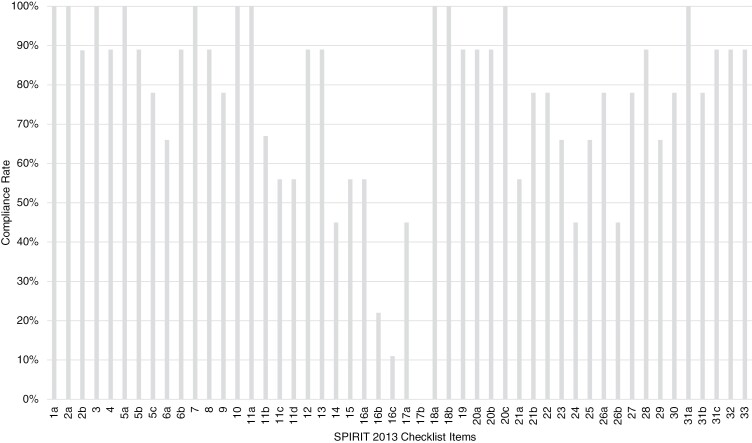
Compliance rate (%) to SPIRIT 2013 statement.

88.8% (*n* = 8) of protocols reported planned statistical analysis and described any planned additional analysis. However, only 77.7% (*n* = 7) of protocols mentioned whether a data monitoring committee (DMC) was present or gave adequate reasons it was not needed. 77.7% (*n* = 7) of protocols explained how personal information about potential and enrolled participants was collected, shared, and maintained in order to protect confidentiality before, during, and after the trial and 88.8% (*n* = 8) of protocols explained who will have access to that data. A summary of the adherence rates for each item in the SPIRIT 2013 checklist can be seen in Figure 2 and in Supplementary Appendix 8.

The included protocols were also analyzed based on the year of publication. A 2-sample *t*-test was performed to compare trials published in 2013–2017 (Group 1, *n* = 5) to trials published between 2018 and 2022 (Group 2, *n* = 4). There was no significant difference in concordance rate (%) between group 1 (mean = 76.8%, *SD*: 0.11) and group 2 (mean = 77.0%, *SD*: 0.06), *P* = .98.

### Quality of Reporting of Clinical Trial Abstracts as per CONSORT-A Checklist

Sixty-eight clinical trial phase II abstracts were assessed against the CONSORT-A statement. Mean adherence rate with the checklist was 67.4% (*SD* ± 0.13). The range of compliance with items from the checklist was 5/17 to 14/17. The total was modified in 94.1% (*n* = 64) of phase II abstracts due to nonapplicable items. Identification of the trial as a phase II trial in the title was present in 88.2% (*n* = 60) of trial abstracts and corresponding author's details were reported in 91.1% (*n* = 62). Adequate trial design was reported in 47.0% (*n* = 32) of included abstracts. Trial hypothesis and objectives were accurately reported in 92.6% (*n* = 63) and 77.9% (*n* = 53) adequately reported the outcome of the trial.

Details on the strategy used for randomization and information on blinding were not applicable in 94.1% (*n* = 64) of phase II abstracts. These trials scored a N/A for these questions on the CONSORT-A checklist to not adversely affect the overall reporting quality percentage. Trial status was reported in 11.8% (*n* = 8) of included abstracts.

**Figure 3.**
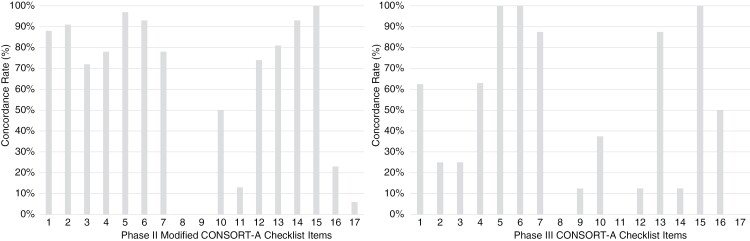
Compliance rate (%) to CONSORT-A checklist items (and modified for analysis of phase II abstracts).

Primary outcome, estimated effect size and precision, conclusions and result interpretation were reported accurately in all abstracts. Details on trial registration and funding were reported poorly in the included abstracts with only 22.0% (*n* = 15) including information on trial registration and only 5.9% (*n* = 4) mentioned trial funding directly in the abstract. A summary of the compliance rates to each item on the CONSORT-A checklist can be seen in Figure 3 and in Supplementary Appendix 9, including modifications made to the CONSORT-A checklist for phase II abstracts.

Eight clinical trial phase III abstracts were assessed against the CONSORT-A statement. Mean modified adherence rate with the checklist was 47.5% (*SD* ± 0.09). The range of compliance with items from the checklist was 6/17 to 11/17. Randomization was identified in the title in 62.5% (*n* = 5) of trial abstracts, while adequate trial design was reported in only 25% (*n* = 2) of included abstracts. All abstracts (*n* = 8) accurately reported the trial objectives and interventions. Trial outcome was reported accurately in 87.5% (*n* = 7) phase III abstracts.

Although trials commonly stated they were randomized, they all failed to discuss the randomization allocation sequence directly in the abstract. This meant that randomization, including the strategy to allocate participants to interventions was inadequately reported in all of the abstracts we assessed. Information on blinding was sub-optimal, with reporting in only 12.5% (*n* = 1) of abstracts.

Trial status was poorly reported in all phase III abstracts (*n* = 0), with no mention of whether the trial was still ongoing, closed to recruitment, or closed to follow up.

Primary outcome, estimated effect size and precision, conclusions and result interpretation were reported accurately in all abstracts (*n* = 8). Details on trial registration and funding were reported poorly in the included abstracts with only 50% (*n* = 4) including information on trial registration and no trial (*n* = 0) mentioned trial funding directly in the abstract. A summary of the compliance rates to each item on the CONSORT-A checklist can be seen in Figure 3 and in Supplementary Appendix 10.

### Quality of Reporting as per CONSORT 2010 Statement

Sixty-eight phase II trials were included in this analysis. After accounting for nonapplicable items, the mean score was 71.3% (*SD* ± 0.10) with a range of 11/27–26/29. Identification of the trial as randomized in the title was present in 88.2% (*n* = 60). All (*n* = 68) included trials discussed the scientific background to their paper and 98.5% (*n* = 67) highlighted any objectives or hypothesis clearly. 82.4% (*n* = 53) of trials discussed the trial design, including allocation ratio, with points only being awarded if both the allocation ratio and design were mentioned. Only 7.4% (*n* = 5) of trials discussed any important changes that were made after the trial commenced. If no changes were made but the trial explicitly stated this, then they received a “Yes”. 98.5% (*n* = 67) described important eligibility criteria for the trial. All trials (*n* = 68) accurately reported the interventions, in sufficient detail to allow replication.

Only 4% of phase II studies were randomized and assessed using an unmodified CONSORT 2010 statement, being scored out of 37 points. The remaining 64 trials were not randomized so were scored “N/A” for questions 8a–11b relating to randomization and blinding. The modified CONSORT 2010 statement for phase II trials is available in Supplementary Appendix 6.

Statistical methods to compare primary and secondary outcomes were reported in 89.8% (*n* = 61) of phase II trials. Similarity of interventions was deemed not applicable for 97.1% (*n* = 66) of included phase II trials because many had interventions that were not comparable. Similarly, 82.4% (*n* = 56) of included phase II trials did not include binary outcomes so were marked as not applicable for this in the CONSORT checklist.

**Figure 4.**
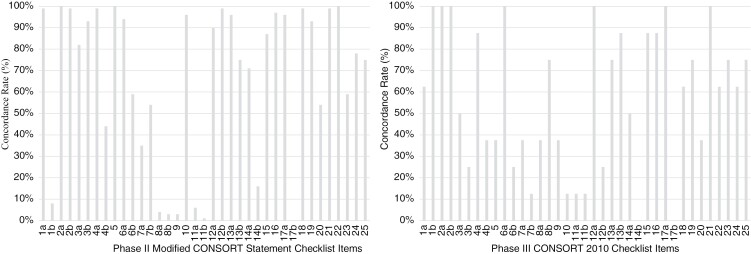
Compliance rate (%) to CONSORT 2010 statement checklist items (and modified for analysis of phase II trials).

Trial generalizability was reported in 98.6% (*n* = 67) included phase II trials. However, the limitations of the trial, including potential sources of bias and misinterpretation, were not as thoroughly reported in only 54.4% (*n* = 37) of the included trials. Only 58.8% of phase II trials (*n* = 40) described the trial registration data and 75% (*n* = 51) described trial funding. The location of the full trial protocol was reported in 77.9% (*n* = 53). A summary of compliance rates can be seen in Figure 4 and in Supplementary Appendix 11.

The included phase II studies were also analyzed based on the year of publication. A 2-sample *t*-test was performed to compare trials published in 2013–2017 (Group 1, *n* = 24) to trials published between 2018 and 2022 (Group 2, *n* = 44). There was a significant difference in concordance rate (%) between Group 1 (mean = 62.9%, *SD*: 0.08) and Group 2 (mean = 75.7%, *SD*: 0.08), *P* = .0001.

Eight phase III trials were included in this analysis. After accounting for nonapplicable items, the mean score was 70.3% (*SD* ± 0.13) with a range of 18/34–25/33. Identification of the trial as randomized in the title was present in 62.5% (*n* = 5). All phase III trials (*n* = 8) discussed the scientific background of their paper and highlighted any objectives or hypothesis clearly. 50.0% (*n* = 4) of trials discussed the trial design, including allocation ratio, with points only being awarded if both the allocation ratio and design were mentioned. Only 25% (*n* = 2) of trials discussed any important changes that were made after the trial commenced. If no changes were made but the trial explicitly stated this, then they would also receive a “Yes” response. All included phase III trials described primary and secondary outcome measures, including how these measures would be assessed. Only 25% (*n* = 2) of phase III trials reported whether changes had been made to the objectives after the trial had commenced. If no changes were made and the trial explicitly stated they would also be given a “Yes” response.

Reporting on randomization methods in the phase III trials was suboptimal. While 62.5% (*n* = 5) of studies identified the trial as randomized in the title, only 37.5% (*n* = 3) described the method used to generate the randomization sequence in the study. The same 37.5% (*n* = 3) trials described the implementation of randomization, including who generated the randomized allocation sequence, who enrolled the participants, and who assigned the interventions. Information on blinding was also inadequate, only being reported in 12.5% (*n* = 1) of studies. If the trial was not randomized any further questions relating to randomization would be scored with a “N/A” to not adversely affect the result.

All the included phase III trials (*n* = 8) described statistical methods used to interpret the results and a further 75% (*n* = 6) explained methods for additional analysis or directly stated no additional analysis was carried out. A table displaying baseline demographic data and clinical characteristics of each group was included in 87.5% (*n* = 7) of the trials.

Trial generalizability was reported in all included trials (*n* = 8). Trial limitations, including sources of bias and misinterpretation, were less well reported in only 37.5% (*n* = 3) of included trials. Descriptions of funding and the trial registration number were included in 75% of the phase III trials (*n* = 6). Only 62.5% (*n* = 5) reported where the full trial protocol could be found. A summary of compliance rates can be seen in Figure 4 and in Supplementary Appendix 12.

Analysis based on the year of publication was not able to be calculated on the included phase III trials due to the limited sample size.

## Discussion

This is the first analysis of the quality of reporting of pediatric neuro-oncology clinical trial protocols and clinical trial results. The study highlights commonalities in reporting deficiencies in pediatric neuro-oncology protocols, abstracts, and trials and suggests that improvements are needed in future publications.

### Trial Protocols and the SPIRIT 2013 Statement

The SPIRIT 2013 statement acts as a checklist of essential components for incorporating into a clinical trial protocol, aimed at promoting thorough and transparent reporting. Throughout the included clinical trial protocols adherence to the SPIRIT statement varied. Among the administrative elements such as title, registration, protocol version, funding, and responsibilities, the adherence to high reporting standards was consistently evident in the included protocols. Additionally, the majority of protocols succeeded in providing an adequate and transparent description of funding sources, thereby enabling a fair assessment of potential conflicts of interest.

While the majority of protocols addressed the research question and provided reasons for conducting the trial, there was a variation in how roles and responsibilities of protocol contributors were conveyed. This included elements such as the structure, functions, and duties of the coordinating center, steering committee, endpoint adjudication committee, data management team, and other individuals or groups supervising the trial. Efforts should be directed towards increasing researchers’ understanding of the vital significance in clearly delineating the composition, roles, and responsibilities of the contributors to the protocol. Each protocol also comprehensively provided information concerning the trial design, thereby enabling the study’s reproducibility by other researchers. This facet plays a pivotal role in grasping the protocol’s contextual framework and confirming the alignment of participants with the stipulated criteria, thus accurately reflecting the intended target demographic.

Eligibility criteria and methodological procedures were well reported throughout the included protocols. Notably, a substantial number of protocols proactively outlined strategies for participant retention and complete follow-up, highlighting their awareness of the significance in maximizing data collection completeness. Furthermore, the statistical methods employed for data analysis, coupled with well-founded justifications for method selection were well reported throughout the included protocols. Equally important, all protocols effectively elucidated strategies to address potential protocol nonadherence and the management of missing data, a pivotal measure to safeguard against bias. Across both the pediatric and adult studies, study methodology and statistical methods were well reported. The comprehensive detailing of the study methodologies in both sets of papers contributes significantly to the transparency and reproducibility of the research.

In line with ethical considerations, a significant number of protocols diligently addressed the requisites of ethical approval and key trial modifications. Acknowledging that ethical approval is a fundamental tenet of clinical research, it’s noteworthy that most protocols fulfilled this obligation. Nevertheless, it’s worth emphasizing the importance of encompassing a detailed account of the ethical approval application process, identifying the granting body, and transparently disclosing any post-approval protocol amendments. This inclusive approach not only enhances the completeness of reporting but also underscores the commitment to ethical integrity within the research process.

However, the area of randomization, which is fundamental to study design, exhibited the poorest reporting throughout the included protocols, warranting particular attention. A minority of protocols offered comprehensive reporting of the methods and mechanisms employed in creating the randomized allocation sequence. The deficiency in reporting randomization details observed in our current study aligns with a recurring trend identified in our previous manuscript assessing adult neuro-oncology protocols.^7^ This underscores a consistent concern that necessitates attention. Randomization plays a foundational role in research trials, and it is imperative that reporting standards are upheld to ensure the method’s robustness and the validity of subsequent analyses. The limited number of protocols that adequately detailed the methods and mechanisms for generating randomized allocation sequences hampers a comprehensive evaluation of treatment effect magnitude, precision, and potential biases. Similarly, only a small proportion of protocols adequately reported details on blinding. When reporting prospective clinical trial protocols, efforts should be made to report succinct and compressive details on the randomization process and, if applicable, blinding.

### Clinical Trial Results and the CONSORT 2010 Statement, Including CONSORT-A

At its core, the CONSORT statement seeks to elevate reporting standards and, consequently, augment the transparency of randomized trials. This is the first analysis of the quality of reporting in pediatric neuro-oncology trials. Within the broader literature, a prior systematic review utilized the CONSORT 2010 statement to evaluate reporting quality in RCTs conducted in head and neck oncology surgery. Similarly to the findings in this study, the systematic review found the mean adherence to the CONSORT checklist to be 45.5% in head and neck oncology articles (*n* = 38). The authors also pinpointed certain deficiencies, notably in the implementation of randomized allocation and the accurate reporting of sample size calculations. These findings underscore the importance of enhancing reporting standards in RCTs not only within neuro-oncology but also across the broader oncological specialty.^9^

To appraise the included abstracts, we utilized the CONSORT-A checklist, a specialized extension of the CONSORT-2010 statement tailored for abstracts. Among the encompassed phase II trials, there was a notable emphasis on reporting the background, aims, objectives, and the foundational research that justified the intervention. The methodology presented in these trials was comprehensive, offering detailed insights into all facets of the trial setup, enabling straightforward replication of the study conditions. In-depth descriptions of trial interventions encompassed crucial aspects, including dosage, administration routes, and procedural intricacies, facilitating effortless replication efforts. This theme was consistent with the included phase III trials, as well as the included trial abstracts where objectives, interventions and outcomes were well reported and in sufficient detail to facilitate replication. The reporting of essential elements of the trial contributes to the robustness and reproducibility of the research, which is fundamental to promote and enforce transparent reporting guidelines.

Sixty-four of the included phase II trials were not randomized. To not adversely affect these trials overall percentage these items were given a “N/A” when being scored. Specifically, items 8a–11b on the CONSORT-2010 checklist were not relevant for these trials, thus the maximum score was decreased to a total of 30. Items 13a and 13b on “participant flow” were also modified to suit nonrandomized trials. In the included phase II trial abstracts checklist items 8 and 9 regarding randomization and blinding respectively were deemed not applicable to 64 of the included abstracts. Checklist item 10 “number of participants randomized to each group” was also modified to the number of participants in each treatment arm, so the checklist item would be relevant to nonrandomized trials.

Randomization and blinding were reported poorly in the included phase III abstracts, with trials consistently failing to describe how the randomization process was carried out in enough detail to score a “Yes” on the CONSORT-A checklist. The inadequacy in reporting randomization persists as a recurring concern, observed consistently across both the previously published adult neuro-oncology report’s assessment of both included protocols and clinical trials, as well as the current pediatric reporting analysis. A consistent pattern emerged, when assessing the included phase III abstracts, with trials inadequately describing the randomization process in sufficient detail to merit a “Yes” score on the CONSORT-A checklist. This trend extended to the included phase III trials, where reporting on randomization continued to be deficient. While many trials delineated the methodology behind generating the randomization allocation sequence, the vast majority failed to describe how this sequence was put into practice. Similarly, the reporting of blinding, a pivotal tool for guarding against potential biases, demonstrated inadequacy in these trials.

The consistent deficiency in reporting randomization and blinding spans both adult and pediatric papers, highlighting the pressing need for improved practices. This issue necessitates awareness-raising, reforms, and meticulous reporting across contexts. Collectively, researchers, authors, and reviewers must address this gap to uphold transparency and rigor in clinical research reporting.

The assessment of findings and interpretations from the included trials was well reported, with a considerable number of trials delving into how their insights resonated with other trial outcomes. This trend remained consistent within the abstracts, where all authors presented a comprehensive overview of their trials’ overarching results. However, a notable gap emerged in the reporting of trial limitations, where only a minority phase II and phase III trials effectively addressed this. It is vital to underscore the importance of a comprehensive discourse on limitations, accompanied by a thorough exploration of methodologies employed to navigate them.

Funding was poorly reported in both the phase II and phase III abstracts analyzed. When assessing the reporting quality of abstracts, funding had to be directly mentioned in the abstract and many authors failed to do this. All of the phase III abstracts failed to report funding sources directly in the abstract. The reporting of funding sources within the abstract of included trials exhibited shortcomings across both the adult and pediatric manuscripts. This deficiency in funding details underscores a common theme between the 2 studies, indicating the need for improved reporting practices for this critical aspect. Ensuring accurate and comprehensive disclosure of funding sources is pivotal for upholding the integrity and impartiality of clinical research, making it imperative to address this issue in efforts to enhance reporting standards moving forward. However, it’s important to note that when examining the full-text papers, funding reporting was generally of a higher standard. This observation highlights the potential impact of complete paper reading in providing a more comprehensive view of funding disclosure practices. It is essential to continue advocating for consistent and transparent reporting of funding sources across all phases of research to maintain research integrity and credibility.

### Limitations

This comprehensive methodological review is based on a sample of neuro-oncology clinical trial literature, which may not represent all studies conducted. The inclusion criteria were limited to articles written in the English language. Despite these limitations, we conducted a thorough search and included common pathologies studied in neuro-oncology clinical trials. To minimize observer bias, 2 review authors (J.S. and S.H.) independently performed data extraction and scoring, achieving a high level of agreement during the initial check. The inclusion of phase II trials and modification of the CONSORT checklist could affect the interpretation of the results in light of the intended purpose of the CONSORT statement; however, because of the small sample of phase III trials in pediatric neuro-oncology, phase II trials are an important research source. The number of included protocols was relatively small, which meant that each article contributed significant weight to the mean percentage adherence score per item. Because of the small sample size, conclusive results cannot be drawn from this. However, this does highlight the infrequency of published clinical trial protocols for this health area. We used the SPIRIT statement (2013), CONSORT, and CONSORT-A statements (2010) post hoc to assess all protocols, and abstracts and trials respectively. As we included only protocols and clinical trial result articles published after 2014, both statements were available for use by authors of the trials. Their use may have been limited due to lack of widespread awareness of these guidelines at time of publication.

## Conclusions

The reporting quality of pediatric neuro-oncology clinical trial protocols and clinical trial result articles is inadequate and requires improvement. Although more than 600 medical journals endorse CONSORT and the list of endorsers of the SPIRIT guidelines is also increasing in size, there needs to be greater awareness and possibly mandatory adherence at the time of manuscript submission, to ensure comprehensive reporting of protocols and clinical trials intended to influence practice. Additionally, societies and cooperative group clinical trials consortia could mandate the use of these guidelines when soliciting abstracts for annual scientific conferences and subsequent publication in specialty journals.

## Supplementary material

Supplementary material is available online at *Neuro-Oncology* (https://academic.oup.com/neuro-oncology).

npae042_suppl_Supplementary_Materials
