# Supplementary material for: Assessing the reporting quality of pediatric neuro-oncology protocols, abstracts, and trials: Adherence to the SPIRIT and CONSORT statements
Source: Neurooncol Pract. 2024 May 11;11(5):617–32. doi: 10.1093/nop/npae042 (PMC11398948; doi:10.1093/nop/npae042)
Supplement: npae042_suppl_Supplementary_Materials [file npae042_suppl_supplementary_materials.docx]

**Supplementary Material**

**PubMed**

| **Database**: PubMed | | |
| --- | --- | --- |
| **Date of search**: 3.3.21 | | |
| Search | Query | Notes |
| #1 | Medulloblastoma*[tiab] |  |
| #2 | Medulloblastoma[Mesh:NoExp] |  |
| #3 | diffuse intrinsic pontine glioma*[tiab] OR diffuse brainstem glioma*[tiab] OR diffuse intrinsic brainstem glioma*[tiab] OR focal brain stem glioma*[tiab] OR midline glioma*[tiab] OR DIPG*[tiab] OR DIBG*[tiab] |  |
| #4 | Diffuse Intrinsic Pontine Glioma[Mesh:NoExp] |  |
| #5 | Ependymoma*[tiab] OR Subependymoma*[tiab] |  |
| #6 | Ependymoma[mesh] |  |
| #7 | Craniopharyngioma*[tiab] |  |
| #8 | Craniopharyngioma[Mesh:NoExp] |  |
| #9 | #1 OR #2 OR #3 OR #4 OR #5 OR #6 OR #7 OR #8 | Common Paediatric tumours |
| #10 | germ cell[tiab] OR germ-cell[tiab] OR embryonal[tiab] OR germinoma*[tiab] |  |
| #11 | Neoplasms, Germ Cell and Embryonal[mesh] |  |
| #12 | #10 OR #11 | Germ cell tumours |
| #13 | intracranial[tiab] OR cranial[tiab] OR brain*[tiab] OR pituitary[tiab] OR pineal[tiab] OR Central nervous system[tiab] OR CNS[tiab] | intracranial |
| #14 | #12 AND #13 | Intracranial germ cell tumours |
| #15 | Grade II Astrocytoma*[tiab] OR Grade 2 Astrocytoma*[tiab] OR Diffuse Astrocytoma*[tiab] OR Fibrillary Astrocytoma*[tiab] OR Protoplasmic Astrocytoma*[tiab] OR Gemistocytic Astrocytoma*[tiab] OR Grade II oligodendroglioma*[tiab] OR Grade 2 oligodendroglioma*[tiab] OR Grade II oligoastrocytoma*[tiab] OR Grade 2 oligoastrocytoma*[tiab] OR Low-grade glioma*[tiab] OR LGG[tiab] OR DLGG[tiab] OR Grade II glioma*[tiab] OR Grade 2 glioma*[tiab] OR Optic nerve glioma*[tiab] OR Subependymal giant cell astrocytoma*[tiab] OR SEGA*[tiab] OR SGCA*[tiab] |  |
| #16 | Astrocytoma[Mesh:NoExp] |  |
| #17 | Oligodendroglioma[Mesh:NoExp] |  |
| #18 | Optic Nerve Glioma[Mesh:NoExp] |  |
| #19 | Grade III Astrocytoma*[tiab] OR Grade 3 Astrocytoma*[tiab] OR Anaplastic astrocytoma*[tiab] OR Grade III oligodendroglioma*[tiab] OR Grade 3 oligodendroglioma*[tiab] OR Anaplastic oligodendroglioma*[tiab] OR Grade III oligoastrocytoma*[tiab] OR Grade 3 oligoastrocytoma*[tiab] OR Anaplastic oligoastrocytoma*[tiab] OR Anaplastic pleomorphic xanthoastrocytoma*[tiab] OR High-grade glioma*[tiab] OR HGG[tiab] OR Malignant glioma*[tiab] OR Grade III glioma*[tiab] OR Grade 3 glioma*[tiab] OR Grade IV glioma*[tiab] OR Grade 4 glioma*[tiab] OR Glioblastoma*[tiab] OR GBM[tiab] |  |
| #20 | Glioblastoma[Mesh:NoExp] |  |
| #21 | #15 OR #16 OR #17 OR #18 OR #19 OR #20 | Low OR High Grade Gliomas |
| #22 | Paediatric*[tiab] OR pediatric*[tiab] OR neonat*[tiab] OR infant*[tiab] OR child*[tiab] OR adolescen*[tiab] OR teen*[tiab] OR young adult*[tiab] |  |
| #23 | Pediatrics[Mesh] OR Infant[Mesh] OR Child[Mesh] OR Adolescent[Mesh:NoExp] OR Young Adult[Mesh:NoExp] |  |
| #24 | #22 OR #23 | Paediatrics |
| #25 | #21 AND #24 | Paediatric Low OR High Grade Gliomas |
| #26 | #9 OR #14 OR #25 | Common Paediatric tumours OR intracranial germ cell tumours OR Paediatric Low OR High Grade Gliomas |
| #27 | Clinical Trials, Phase II as Topic[Mesh] |  |
| #28 | Clinical Trials, Phase III as Topic[Mesh] |  |
| #29 | Clinical Trials, Phase IV as Topic[Mesh] |  |
| #30 | random allocation[mh] |  |
| #31 | double-blind method[mh] |  |
| #32 | single-blind method[mh] |  |
| #33 | placebos[mh] |  |
| #34 | randomized controlled trial[pt] |  |
| #35 | controlled clinical trial[pt] |  |
| #36 | clinical trial, phase ii[pt] |  |
| #37 | clinical trial, phase iii[pt] |  |
| #38 | clinical trial, phase iv[pt] |  |
| #39 | clinical trial*[tiab] |  |
| #40 | control trial*[tiab] |  |
| #41 | controlled trial*[tiab] |  |
| #42 | randomi*[tiab] |  |
| #43 | single blind*[tiab] |  |
| #44 | double blind*[tiab] |  |
| #45 | triple blind*[tiab] |  |
| #46 | treble blind*[tiab] |  |
| #47 | (phase II[tiab] OR phase 2[tiab]) |  |
| #48 | (phase III[tiab] OR phase 3[tiab]) |  |
| #49 | (phase IV[tiab] OR phase 4[tiab]) |  |
| #50 | placebo*[tiab] |  |
| #51 | #27 OR #28 OR #29 OR #30 OR #31 OR #32 OR #33 OR #34 OR #35 OR #36 OR #37 OR #38 OR #39 OR #40 OR #41 OR #42 OR #43 OR #44 OR #45 OR #46 OR #47 OR #48 OR #49 OR #50 | Clinical trials |
| #52 | #26 AND #51 | 4,754 |
| #53 | #52 was searched. English language filter was applied.  Humans  Filters applied: English, Humans, from 01/01/2000 – 31/12/2014 | **2,140** |

**EMBASE**

| **Database**: Embase | | |
| --- | --- | --- |
| **Date of search**: 3.3.21 | | |
| Search | Query | Items found |
| 1 | ‘Medulloblastoma*’:ti,ab |  |
| 2 | ‘Medulloblastoma’/de |  |
| 3 | (‘diffuse intrinsic pontine glioma*’ OR ‘diffuse brainstem glioma*’ OR ‘diffuse intrinsic brainstem glioma*’ OR ‘focal brain stem glioma*’ OR ‘midline glioma*’ OR ‘DIPG*’ OR ‘DIBG*’):ti,ab |  |
| 4 | ‘pontine glioma’/de |  |
| 5 | (‘Ependymoma*’ OR ‘Subependymoma*’):ti,ab |  |
| 6 | ‘Ependymoma’/exp |  |
| 7 | ‘Craniopharyngioma*’:ti,ab |  |
| 8 | ‘Craniopharyngioma’/de |  |
| 9 | #1 OR #2 OR #3 OR #4 OR #5 OR #6 OR #7 OR #8 | Common tumours |
| 10 | (‘germ cell’ OR ‘germ-cell’ OR ‘embryonal’ OR ‘germinoma*’):ti,ab |  |
| 11 | ‘germ cell cancer’/exp |  |
| 12 | #10 OR #11 | Germ cell |
| 13 | (‘intracranial’ OR ‘cranial’ OR ‘brain*’ OR ‘pituitary’ OR ‘pineal’ OR ‘Central nervous system’ OR ‘CNS’):ti,ab | intracranial |
| 14 | #12 AND #13 | Intracranial germ cell |
| 15 | (‘Grade II Astrocytoma*’ OR ‘Grade 2 Astrocytoma*’ OR ‘Diffuse Astrocytoma*’ OR ‘Fibrillary Astrocytoma*’ OR ‘Protoplasmic Astrocytoma*’ OR ‘Gemistocytic Astrocytoma*’ OR ‘Grade II oligodendroglioma*’ OR ‘Grade 2 oligodendroglioma*’ OR ‘Grade II oligoastrocytoma*’ OR ‘Grade 2 oligoastrocytoma*’ OR ‘Low-grade glioma*’ OR ‘LGG’ OR ‘DLGG’ OR ‘Grade II glioma*’ OR ‘Grade 2 glioma*’ OR ‘Optic nerve glioma*’ OR ‘Subependymal giant cell astrocytoma*’ OR ‘SEGA*’ OR ‘SGCA*’):ti,ab |  |
| 16 | ‘Astrocytoma’/de |  |
| 17 | ‘Oligodendroglioma’/de |  |
| 18 | ‘Optic Nerve Glioma’/de |  |
| 19 | (‘Grade III Astrocytoma*’ OR ‘Grade 3 Astrocytoma*’ OR ‘Anaplastic astrocytoma*’ OR ‘Grade III oligodendroglioma*’ OR ‘Grade 3 oligodendroglioma*’ OR ‘Anaplastic oligodendroglioma*’ OR ‘Grade III oligoastrocytoma*’ OR ‘Grade 3 oligoastrocytoma*’ OR ‘Anaplastic oligoastrocytoma*’ OR ‘Anaplastic pleomorphic xanthoastrocytoma*’ OR ‘High-grade glioma*’ OR ‘HGG’ OR ‘Malignant glioma*’ OR ‘Grade III glioma*’ OR ‘Grade 3 glioma*’ OR ‘Grade IV glioma*’ OR ‘Grade 4 glioma*’ OR ‘Glioblastoma*’ OR ‘GBM’):ti,ab |  |
| 20 | ‘Glioblastoma’/de |  |
| 21 | #15 OR #16 OR #17 OR #18 OR #19 OR #20 | Gliomas |
| 22 | (‘Paediatric*’ OR ‘pediatric*’ OR ‘neonat*’ OR ‘infant*’ OR ‘child*’ OR ‘adolescen*’ OR ‘teen*’ OR ‘young adult*’):ti,ab |  |
| 23 | ‘pediatrics’/exp OR ‘juvenile’/exp OR ‘Young Adult’/de |  |
| 24 | #22 OR #23 | Paediatrics |
| 25 | #21 AND #24 | Paediatric gliomas |
| 26 | #9 OR #14 OR #25 | All tumours |
| 27 | 'phase 2 clinical trial'/de |  |
| 28 | 'phase 3 clinical trial'/de |  |
| 29 | 'phase 4 clinical trial'/de |  |
| 30 | 'randomization'/exp |  |
| 31 | 'double blind procedure'/de |  |
| 32 | 'single blind procedure'/de |  |
| 33 | 'placebo'/de |  |
| 34 | ‘clinical trial*’:ti,ab |  |
| 35 | ‘control trial*’:ti,ab |  |
| 36 | ‘controlled trial*’:ti,ab |  |
| 37 | ‘randomi*’:ti,ab |  |
| 38 | ((singl* or doubl* or tripl* or treb*) NEAR/25 (blind* or mask*)):ti,ab |  |
| 39 | ‘phase II’:ti,ab |  |
| 40 | ‘phase 2’:ti,ab |  |
| 41 | ‘phase III’:ti,ab |  |
| 42 | ‘phase 3’:ti,ab |  |
| 43 | ‘phase IV’:ti,ab |  |
| 44 | ‘phase 4’:ti,ab |  |
| 45 | ‘placebo*’:ti,ab |  |
| 46 | #27 OR #28 OR #29 OR #30 OR #31 OR #32 OR #33 OR #34 OR #35 OR #36 OR #37 OR #38 OR #39 OR #40 OR #41 OR #42 OR #43 OR #44 OR #45 | trials |
| 47 | #26 AND #46 | 3,939 |
| 48 | #26 AND #46 AND [humans]/lim AND [english]/lim AND [2000-2014]/py | **1,520** |

**CINAHL**

| **Database**: CINAHL (EBSCO Host) | | |
| --- | --- | --- |
| **Date of search**: 4.3.21 | | |
| Search | Query | Items found |
| S1 | TI Medulloblastoma* |  |
| S2 | AB Medulloblastoma* |  |
| S3 | TI diffuse intrinsic pontine glioma* OR diffuse brainstem glioma* OR diffuse intrinsic brainstem glioma* OR focal brain stem glioma* OR midline glioma* OR DIPG* OR DIBG* |  |
| S4 | AB diffuse intrinsic pontine glioma* OR diffuse brainstem glioma* OR diffuse intrinsic brainstem glioma* OR focal brain stem glioma* OR midline glioma* OR DIPG* OR DIBG* |  |
| S5 | MH “Diffuse Intrinsic Pontine Glioma” |  |
| S6 | TI Ependymoma* OR Subependymoma* |  |
| S7 | AB Ependymoma* OR Subependymoma* |  |
| S8 | TI Craniopharyngioma* |  |
| S9 | AB Craniopharyngioma* |  |
| S10 | MH “Craniopharyngioma” |  |
| S11 | S1 OR S2 OR S3 OR S4 OR S5 OR S6 OR S7 OR S8 OR S9 OR S10 | Common Paediatric tumours |
| S12 | TI germ cell OR germ-cell OR embryonal OR germinoma* |  |
| S13 | AB germ cell OR germ-cell OR embryonal OR germinoma* |  |
| S14 | MH “Neoplasms, Germ Cell and Embryonal+” |  |
| S15 | S12 OR S13 OR S14 | Germ cell tumours |
| S16 | TI intracranial OR cranial OR brain* OR pituitary OR pineal OR Central nervous system OR CNS |  |
| S17 | AB intracranial OR cranial OR brain* OR pituitary OR pineal OR Central nervous system OR CNS |  |
| S18 | S16 OR S17 | intracranial |
| S19 | S15 AND S18 | Intracranial germ cell tumours |
| S20 | TI Grade II Astrocytoma* OR Grade 2 Astrocytoma* OR Diffuse Astrocytoma* OR Fibrillary Astrocytoma* OR Protoplasmic Astrocytoma* OR Gemistocytic Astrocytoma* OR Grade II oligodendroglioma* OR Grade 2 oligodendroglioma* OR Grade II oligoastrocytoma* OR Grade 2 oligoastrocytoma* OR Low-grade glioma* OR LGG OR DLGG OR Grade II glioma* OR Grade 2 glioma* OR Optic nerve glioma* OR Subependymal giant cell astrocytoma* OR SEGA* OR SGCA* |  |
| S21 | AB Grade II Astrocytoma* OR Grade 2 Astrocytoma* OR Diffuse Astrocytoma* OR Fibrillary Astrocytoma* OR Protoplasmic Astrocytoma* OR Gemistocytic Astrocytoma* OR Grade II oligodendroglioma* OR Grade 2 oligodendroglioma* OR Grade II oligoastrocytoma* OR Grade 2 oligoastrocytoma* OR Low-grade glioma* OR LGG OR DLGG OR Grade II glioma* OR Grade 2 glioma* OR Optic nerve glioma* OR Subependymal giant cell astrocytoma* OR SEGA* OR SGCA* |  |
| S22 | MH “Glioma” |  |
| S23 | TI Grade III Astrocytoma* OR Grade 3 Astrocytoma* OR Anaplastic astrocytoma* OR Grade III oligodendroglioma* OR Grade 3 oligodendroglioma* OR Anaplastic oligodendroglioma* OR Grade III oligoastrocytoma* OR Grade 3 oligoastrocytoma* OR Anaplastic oligoastrocytoma* OR Anaplastic pleomorphic xanthoastrocytoma* OR High-grade glioma* OR HGG OR Malignant glioma* OR Grade III glioma* OR Grade 3 glioma* OR Grade IV glioma* OR Grade 4 glioma* OR Glioblastoma* OR GBM |  |
| S24 | AB Grade III Astrocytoma* OR Grade 3 Astrocytoma* OR Anaplastic astrocytoma* OR Grade III oligodendroglioma* OR Grade 3 oligodendroglioma* OR Anaplastic oligodendroglioma* OR Grade III oligoastrocytoma* OR Grade 3 oligoastrocytoma* OR Anaplastic oligoastrocytoma* OR Anaplastic pleomorphic xanthoastrocytoma* OR High-grade glioma* OR HGG OR Malignant glioma* OR Grade III glioma* OR Grade 3 glioma* OR Grade IV glioma* OR Grade 4 glioma* OR Glioblastoma* OR GBM |  |
| S25 | S20 OR S21 OR S22 OR S23 OR S24 | Low OR High Grade Gliomas |
| S26 | TI Paediatric* OR pediatric* OR neonat* OR infant* OR child* OR adolescen* OR teen* OR young adult* |  |
| S27 | AB Paediatric* OR pediatric* OR neonat* OR infant* OR child* OR adolescen* OR teen* OR young adult* |  |
| S28 | MH “Child+” |  |
| S29 | MH “[Adolescence](https://web-b-ebscohost-com.liverpool.idm.oclc.org/ehost/mesh/tree?term=Adolescence&sid=23626650-8fb9-4e2a-adfc-47d3ca5060ae%40pdc-v-sessmgr03&vid=31)+” |  |
| S30 | MH “Young Adult” |  |
| S31 | S26 OR S27 OR S28 OR S29 OR S30 | Paediatrics |
| S32 | S25 AND S31 | Paediatric Low OR High Grade Gliomas |
| S33 | S11 OR S19 OR S32 | Common Paediatric tumours OR intracranial germ cell tumours OR Paediatric Low OR High Grade Gliomas |
| S34 | MH "Clinical Trials+" |  |
| S35 | MH "Random Sample+" |  |
| S36 | MH "Placebos" |  |
| S37 | PT randomized controlled trial |  |
| S38 | PT clinical trial |  |
| S39 | TI clinical trial* OR AB clinical trial* |  |
| S40 | TI control* trial* OR AB control* trial* |  |
| S41 | TI randomi* OR AB randomi* |  |
| S42 | TI ((singl* OR doubl* OR tripl* OR treb*) AND (blind* OR mask*)) |  |
| S43 | AB ((singl* OR doubl* OR tripl* OR treb*) AND (blind* OR mask*)) |  |
| S44 | TI (phase II OR phase III OR phase IV OR phase 2 OR phase 3 OR phase 4) |  |
| S45 | AB (phase II OR phase III OR phase IV OR phase 2 OR phase 3 OR phase 4) |  |
| S46 | TI placebo* OR AB placebo* |  |
| S47 | S34 OR S35 OR S36 OR S37 OR S38 OR S39 OR S40 OR S41 OR S42 OR S43 OR S44 OR S45 OR S46 |  |
| S48 | S33 AND S47 | 1,611 |
| S49 | S33 & S47  Limiters - Publication Year: 2000-2014; English Language; Human | 581 |

**Cochrane central register of controlled trials**

| **Database**: Cochrane central register of controlled trials | | | |
| --- | --- | --- | --- |
| **Date of search**: 4.3.21 | | | |
| Search | Query | Notes | Other |
| #1 | Medulloblastoma* | Title Abstract Keyword |  |
| #2 | Medulloblastoma | MeSH term- this term only |  |
| #3 | diffuse intrinsic pontine glioma* OR diffuse brainstem glioma* OR diffuse intrinsic brainstem glioma* OR focal brain stem glioma* OR midline glioma* OR DIPG* OR DIBG* | Title Abstract Keyword |  |
| #4 | Diffuse Intrinsic Pontine Glioma | MeSH term- this term only |  |
| #5 | Ependymoma* OR Subependymoma* | Title Abstract Keyword |  |
| #6 | Ependymoma | MeSH term- explode all trees |  |
| #7 | Craniopharyngioma* | Title Abstract Keyword |  |
| #8 | Craniopharyngioma | MeSH term- this term only |  |
| #9 | #1 OR #2 OR #3 OR #4 OR #5 OR #6 OR #7 OR #8 |  | Common Paediatric tumours |
| #10 | germ cell OR germ-cell OR embryonal OR germinoma* | Title Abstract Keyword |  |
| #11 | Neoplasms, Germ Cell and Embryonal | MeSH term- explode all trees |  |
| #12 | #10 OR #11 |  | Germ cell tumours |
| #13 | intracranial OR cranial OR brain* OR pituitary OR pineal OR Central nervous system OR CNS | Title Abstract Keyword | intracranial |
| #14 | #12 AND #13 |  | Intracranial germ cell tumours |
| #15 | Grade II Astrocytoma* OR Grade 2 Astrocytoma* OR Diffuse Astrocytoma* OR Fibrillary Astrocytoma* OR Protoplasmic Astrocytoma* OR Gemistocytic Astrocytoma* OR Grade II oligodendroglioma* OR Grade 2 oligodendroglioma* OR Grade II oligoastrocytoma* OR Grade 2 oligoastrocytoma* OR Low-grade glioma* OR LGG OR DLGG OR Grade II glioma* OR Grade 2 glioma* OR Optic nerve glioma* OR Subependymal giant cell astrocytoma* OR SEGA* OR SGCA* | Title Abstract Keyword |  |
| #16 | Astrocytoma | MeSH term- this term only |  |
| #17 | Oligodendroglioma | MeSH term- this term only |  |
| #18 | Optic Nerve Glioma | MeSH term- this term only |  |
| #19 | Grade III Astrocytoma* OR Grade 3 Astrocytoma* OR Anaplastic astrocytoma* OR Grade III oligodendroglioma* OR Grade 3 oligodendroglioma* OR Anaplastic oligodendroglioma* OR Grade III oligoastrocytoma* OR Grade 3 oligoastrocytoma* OR Anaplastic oligoastrocytoma* OR Anaplastic pleomorphic xanthoastrocytoma* OR High-grade glioma* OR HGG OR Malignant glioma* OR Grade III glioma* OR Grade 3 glioma* OR Grade IV glioma* OR Grade 4 glioma* OR Glioblastoma* OR GBM | Title Abstract Keyword |  |
| #20 | Glioblastoma | MeSH term- this term only |  |
| #21 | #15 OR #16 OR #17 OR #18 OR #19 OR #20 |  | Low OR High Grade Gliomas |
| #22 | Paediatric* OR pediatric* OR neonat* OR infant* OR child* OR adolescen* OR teen* OR young adult* | Title Abstract Keyword |  |
| #23 | Pediatrics | MeSH term- explode all trees |  |
| #24 | Infant | MeSH term- explode all trees |  |
| #25 | Child | MeSH term- explode all trees |  |
| #26 | Adolescent | MeSH term- this term only |  |
| #27 | Young Adult | MeSH term- this term only |  |
| #28 | #22 OR #23 OR #24 OR #25 OR #26 OR #27 |  | Paediatrics |
| #29 | #21 AND #28 |  | Paediatric Low OR High Grade Gliomas |
| #30 | #9 OR #14 OR #29 |  | Common Paediatric tumours OR intracranial germ cell tumours OR Paediatric Low OR High Grade Gliomas |
| #31 | Clinical Trial, Phase II | MeSH term- this term only |  |
| #32 | Clinical Trial, Phase III | MeSH term- this term only |  |
| #33 | Clinical Trial, Phase IV | MeSH term- this term only |  |
| #34 | Random allocation | MeSH term this term only |  |
| #35 | Double-blind method | MeSH term this term only |  |
| #36 | Single-blind method | MeSH term this term only |  |
| #37 | Placebos | MeSH term this term only |  |
| #38 | clinical trial* | Title Abstract Keyword |  |
| #39 | control trial* | Title Abstract Keyword |  |
| #40 | controlled trial* | Title Abstract Keyword |  |
| #41 | randomi* | Title Abstract Keyword |  |
| #42 | (singl* or doubl* or tripl* or treb*) NEAR/25 (blind* or mask*) | Title Abstract Keyword |  |
| #43 | phase II OR phase 2 | Title Abstract Keyword |  |
| #44 | phase III OR phase 3 | Title Abstract Keyword |  |
| #45 | phase IV OR phase 4 | Title Abstract Keyword |  |
| #46 | placebo* | Title Abstract Keyword |  |
| #47 | #31 OR #32 OR #33 OR #34 OR #35 OR #36 OR #37 OR #38 OR #39 OR #40 OR #41 OR #42 OR #43 OR #44 OR #45 OR #46 |  | Terminology related to trials |
| #48 | #30 AND #47 |  | 1,520 |
| #49 | **Trials filter selected**  **with Cochrane Library publication date (year first published) from 2000 to 2014** |  | **638** |

*Supplementary Appendix 1: Full electronic search strategy*

|  | **Inclusion** | **Exclusion** |
| --- | --- | --- |
| **Population** | - Children and young adults | - Adults |
| **Intervention** | - Perioperative care - Surgery - Radiotherapy - Radiosurgery - Medical therapy - Active monitoring - Combinations of the above |  |
| **Condition** | - Paediatric brain tumours - Medulloblastoma - Glioma (of any kind including low-grade, high-grade, DIPG. These have a range of names e.g. astrocytoma, oligodendroglioma- separate file attached) - Ependymoma - Craniopharyngioma - Cranial germ-cell tumours, embryonal tumours | - Primary spinal tumours |
| **Study type** | - Phase III trials - Primary research - English language - Full text available - Studies including 10 or more patients | - Systematic reviews, meta-analyses - Animal studies |

*Supplementary Appendix 2: Inclusion and exclusion criteria*

| **Checklist Item** | **Item Number** | **Description** | **Additional Guidance** |
| --- | --- | --- | --- |
| **Administrative Information** | | | |
| Title | 1 | Descriptive title identifying the study design, population, interventions, and, if applicable, trial acronym |  |
| Trial Registration | 2a | Trial identifier and registry name. If not yet registered, name of intended registry |  |
|  | 2b | All items from the World Health Organization Trial Registration Data Set |  |
| Protocol version | 3 | Date and version identifier |  |
| Funding | 4 | Sources and types of financial, material, and other support |  |
| Roles and Responsibilities | 5a | Names, affiliations, and roles of protocol contributors |  |
|  | 5b | Name and contact information for the trial sponsor |  |
|  | 5c | Role of study sponsor and funders, if any, in study design; collection, management, analysis, and interpretation of data; writing of the report; and the decision to submit the report for publication, including whether they will have ultimate authority over any of these activities |  |
|  | 5d | Composition, roles, and responsibilities of the coordinating centre, steering committee, endpoint adjudication committee, data management team, and other individuals or groups overseeing the trial, if applicable (see Item 21a for data monitoring committee) |  |
| **Introduction** | | | |
| Background and Rationale | 6a | Description of research question and justification for undertaking the trial, including summary of relevant studies (published and unpublished) examining benefits and harms for each intervention |  |
|  | 6b | Explanation for choice of comparators |  |
| Objectives | 7 | Specific objectives or hypotheses |  |
| Trial Design | 8 | Description of trial design including type of trial (eg, parallel group, crossover, factorial, single group), allocation ratio, and framework (eg, superiority, equivalence, noninferiority, exploratory) |  |
| **Methods: Participants, interventions, and outcomes** | | | |
| Study Setting | 9 | Description of study settings (eg, community clinic, academic hospital) and list of countries where data will be collected. Reference to where list of study sites can be obtained |  |
| Eligibility Criteria | 10 | Inclusion and exclusion criteria for participants. If applicable, eligibility criteria for study centres and individuals who will perform the interventions (eg, surgeons, psychotherapists) |  |
| Interventions | 11a | Interventions for each group with sufficient detail to allow replication, including how and when they will be administered |  |
|  | 11b | Criteria for discontinuing or modifying allocated interventions for a given trial participant (eg, drug dose change in response to harms, participant request, or improving/worsening disease) |  |
|  | 11c | Strategies to improve adherence to intervention protocols, and any procedures for monitoring adherence (eg, drug tablet return, laboratory tests) |  |
|  | 11d | Relevant concomitant care and interventions that are permitted or prohibited during the trial |  |
| Outcomes | 12 | Primary, secondary, and other outcomes, including the specific measurement variable (eg, systolic blood pressure), analysis metric (eg, change from baseline, final value, time to event), method of aggregation (eg, median, proportion), and time point for each outcome. Explanation of the clinical relevance of chosen efficacy and harm outcomes is strongly recommended |  |
| Participant Timeline | 13 | Time schedule of enrolment, interventions (including any run-ins and washouts), assessments, and visits for participants. A schematic diagram is highly recommended (see Figure) |  |
| Sample Size | 14 | Estimated number of participants needed to achieve study objectives and how it was determined, including clinical and statistical assumptions supporting any sample size calculations |  |
| Recruitment | 15 | Strategies for achieving adequate participant enrolment to reach target sample size |  |
| **Methods: Assignment of interventions (for controlled trials)** | | | |
| Allocation: | | | |
| Sequence Generation | 16a | Method of generating the allocation sequence (eg, computer-generated random numbers), and list of any factors for stratification. To reduce predictability of a random sequence, details of any planned restriction (eg, blocking) should be provided in a separate document that is unavailable to those who enrol participants or assign interventions |  |
| Allocation Concealment Mechanism | 16b | Mechanism of implementing the allocation sequence (eg, central telephone; sequentially numbered, opaque, sealed envelopes), describing any steps to conceal the sequence until interventions are assigned |  |
| Implementation | 16c | Who will generate the allocation sequence, who will enrol participants, and who will assign participants to interventions |  |
| Blinding (Masking) | 17a | Who will be blinded after assignment to interventions (eg, trial participants, care providers, outcome assessors, data analysts), and how |  |
|  | 17b | If blinded, circumstances under which unblinding is permissible, and procedure for revealing a participant’s allocated intervention during the trial |  |
| **Methods: Data collection, management, and analysis** | | | |
| Data Collection Methods | 18a | Plans for assessment and collection of outcome, baseline, and other trial data, including any related processes to promote data quality (eg, duplicate measurements, training of assessors) and a description of study instruments (eg, questionnaires, laboratory tests) along with their reliability and validity, if known. Reference to where data collection forms can be found, if not in the protocol |  |
|  | 18b | Plans to promote participant retention and complete follow-up, including list of any outcome data to be collected for participants who discontinue or deviate from intervention protocols |  |
| Data Management | 19 | Plans for data entry, coding, security, and storage, including any related processes to promote data quality (eg, double data entry; range checks for data values). Reference to where details of data management procedures can be found, if not in the protocol |  |
| Statistical Methods | 20a | Statistical methods for analysing primary and secondary outcomes. Reference to where other details of the statistical analysis plan can be found, if not in the protocol |  |
|  | 20b | Methods for any additional analyses (eg, subgroup and adjusted analyses) |  |
|  | 20c | Definition of analysis population relating to protocol non-adherence (eg, as randomised analysis), and any statistical methods to handle missing data (eg, multiple imputation) |  |
| **Methods: Monitoring** | | | |
| Data Monitoring | 21a | Composition of data monitoring committee (DMC); summary of its role and reporting structure; statement of whether it is independent from the sponsor and competing interests; and reference to where further details about its charter can be found, if not in the protocol. Alternatively, an explanation of why a DMC is not needed |  |
|  | 21b | Description of any interim analyses and stopping guidelines, including who will have access to these interim results and make the final decision to terminate the trial |  |
| Harms | 22 | Plans for collecting, assessing, reporting, and managing solicited and spontaneously reported adverse events and other unintended effects of trial interventions or trial conduct |  |
| Auditing | 23 | Frequency and procedures for auditing trial conduct, if any, and whether the process will be independent from investigators and the sponsor |  |
| **Ethics and Dissemination** | | | |
| Research ethics approval | 24 | Plans for seeking research ethics committee/institutional review board (REC/IRB) approval |  |
| Protocol amendments | 25 | Plans for communicating important protocol modifications (eg, changes to eligibility criteria, outcomes, analyses) to relevant parties (eg, investigators, REC/IRBs, trial participants, trial registries, journals, regulators) |  |
| Consent or assent | 26a | Who will obtain informed consent or assent from potential trial participants or authorised surrogates, and how (see Item 32) |  |
|  | 26b | Additional consent provisions for collection and use of participant data and biological specimens in ancillary studies, if applicable |  |
| Confidentiality | 27 | How personal information about potential and enrolled participants will be collected, shared, and maintained in order to protect confidentiality before, during, and after the trial |  |
| Declaration of interests | 28 | Financial and other competing interests for principal investigators for the overall trial and each study site |  |
| Access to data | 29 | Statement of who will have access to the final trial dataset, and disclosure of contractual agreements that limit such access for investigators |  |
| Ancillary and post-trial care | 30 | Provisions, if any, for ancillary and post-trial care, and for compensation to those who suffer harm from trial participation |  |
| Dissemination policy | 31a | Plans for investigators and sponsor to communicate trial results to participants, healthcare professionals, the public, and other relevant groups (eg, via publication, reporting in results databases, or other data sharing arrangements), including any publication restrictions |  |
|  | 31b | Authorship eligibility guidelines and any intended use of professional writers |  |
|  | 31c | Plans, if any, for granting public access to the full protocol, participant-level dataset, and statistical code |  |
| **Appendices** | | | |
| Informed Consent Materials | 32 | Model consent form and other related documentation given to participants and authorised surrogates |  |
| Biological Specimens | 33 | Plans for collection, laboratory evaluation, and storage of biological specimens for genetic or molecular analysis in the current trial and for future use in ancillary studies, if applicable |  |

*Supplementary Appendix 3: SPIRIT 2013 checklist, with additional guidance to aid analysis*

| **Checklist Item** | **Item Number** | **Description** | **Additional Guidance** |
| --- | --- | --- | --- |
| **Title** | 1* | Identification of the study as a trial (inc. phase of the trial) | This item has been modified for analysis of phase II trial abstracts |
| **Authors** | 2 | Contact details for the corresponding author |  |
| **Trial Design** | 3 | Description of the trial design (e.g. parallel, cluster, non-inferiority) |  |
| **Methods** | | | |
| Participants | 4 | Eligibility criteria for participants and the settings where the data were collected | Cannot just mention that there are eligibility criteria, must also state what the criteria are |
| Interventions | 5 | Interventions intended for each group |  |
| Objective | 6 | Specific objective or hypothesis |  |
| Outcome | 7 | Clearly defined primary outcome for this report |  |
| Randomisation* | 8 | How participants were allocated to interventions | If no randomisation has taken place (unrandomized trial) this item is graded ‘N/A’ |
| Blinding (Masking)* | 9 | Whether or not participants, care givers, and those assessing the outcomes were blinded to group assignment | If no blinding has taken place this item is graded ‘N/A’ |
| **Results** | | | |
| Numbers Randomised* | 10 | Number of participants in each group | This item has been modified for analysis of phase II trial abstracts |
| Recruitment | 11 | Trial Status |  |
| Numbers Analysed | 12 | Number of participants analysed in each group |  |
| Outcome | 13 | For the primary outcome, a result for each group and the estimated effect size and its precision |  |
| Harms | 14 | Important adverse events or side effects |  |
| **Conclusions** | 15 | General interpretation of the results |  |
| **Trial Registration** | 16 | Registration number and name of trial register | Answer ‘YES’ only if mentioned in abstract – If in full article only, answer ‘NO’ |
| **Funding** | 17 | Source of funding | Answer ‘YES’ only if mentioned in abstract – If in full article only, answer ‘NO’ |

*Supplementary Table 4: Modified CONSORT-A 2010, with additional guidance to aid analysis of phase II trial abstracts*

| **Checklist Item** | **Item Number** | **Description** | **Additional Guidance** |
| --- | --- | --- | --- |
| **Title** | 1 | Identification of the study as randomized |  |
| **Authors** | 2 | Contact details for the corresponding author |  |
| **Trial Design** | 3 | Description of the trial design (e.g. parallel, cluster, non-inferiority) |  |
| **Methods** | | | |
| Participants | 4 | Eligibility criteria for participants and the settings where the data were collected | Cannot just mention that there are eligibility criteria, must also state what the criteria are. |
| Interventions | 5 | Interventions intended for each group |  |
| Objective | 6 | Specific objective or hypothesis |  |
| Outcome | 7 | Clearly defined primary outcome for this report |  |
| Randomisation | 8 | How participants were allocated to interventions |  |
| Blinding (Masking) | 9 | Whether or not participants, care givers, and those assessing the outcomes were blinded to group assignment |  |
| **Results** | | | |
| Numbers Randomised | 10 | Number of participants randomized to each group |  |
| Recruitment | 11 | Trial Status |  |
| Numbers Analysed | 12 | Number of participants analysed in each group |  |
| Outcome | 13 | For the primary outcome, a result for each group and the estimated effect size and its precision |  |
| Harms | 14 | Important adverse events or side effects |  |
| **Conclusions** | 15 | General interpretation of the results |  |
| **Trial Registration** | 16 | Registration number and name of trial register |  |
| **Funding** | 17 | Source of funding |  |

*Supplementary Table 5: CONSORT-A 2010 checklist, with additional guidance to aid analysis*

| **Section/Topic** | **Item Number** | **Item Description** | **Additional Guidance** |
| --- | --- | --- | --- |
| **Title and Abstract** | | | |
|  | 1a* | Identification as a trial in the title (inc. phase of the trial) | This item has been modified for analysis of phase II trials |
|  | 1b | Structured summary of trial design, methods, results, and conclusions | If it has a “structured summary of trial design, methods, results and conclusions”, it can be reported as YES. An in- depth analysis of the abstract is done separately using CONSORT-A. |
| **Introduction** | | | |
| Background and Objectives | 2a | Scientific background and explanation of  rationale |  |
|  | 2b | Specific objectives or hypotheses |  |
| **Methods** | | | |
|  | 3a | Description of trial design (such as parallel, factorial) including allocation ratio |  |
|  | 3b | Important changes to methods after trial commencement (such as eligibility criteria), with reasons |  |
| Participants | 4a | Eligibility criteria for participants collected | Cannot just mention that there are eligibility criteria, must also state what the criteria are. |
|  | 4b | Settings and locations where the data were collected |  |
| Interventions | 5 | The interventions for each group with sufficient details to allow replication, including how and when they were actually administered |  |
| Outcomes | 6a | Completely defined pre-specified primary and secondary outcome measures, including how and when they were assessed |  |
|  | 6b | Any changes to trial outcomes after the trial commenced, with reasons |  |
| Sample Size | 7a | How sample size was determined |  |
|  | 7b | When applicable, explanation of interim analyses and stopping guidelines |  |
| Randomisation: 8a to 11b are only relevant if the study is randomised – Otherwise score ‘N/A’ | | | |
| Sequence Generation | 8a* | Method used to generate the random  allocation sequence |  |
|  | 8b* | Type of randomisation; details of any  restriction (such as blocking and block size) |  |
| Allocation concealment mechanism | 9* | Mechanism used to implement the random allocation sequence (such as sequentially numbered containers), describing any steps taken to conceal the sequence until interventions were assigned |  |
| Implementation | 10* | Who generated the random allocation sequence, who enrolled participants, and who assigned participants to interventions |  |
| Blinding | 11a* | If done, who was blinded after assignment to interventions (for example, participants, care providers, those assessing outcomes) and how |  |
|  | 11b* | If relevant, description of the similarity of interventions | Not applicable in all trials. |
| Statistical Methods | 12a | Statistical methods used to compare groups for primary and secondary outcomes |  |
|  | 12b | Methods for additional analyses, such as subgroup analyses and adjusted analyses |  |
| **Results** | | | |
| Participant flow (diagram strongly recommended) | 13a* | For each group, the numbers of participants who were randomly/otherwise assigned, received intended treatment, and were analysed for the primary outcome | This item has been modified for analysis of phase II trials |
|  | 13b* | For each group, losses and exclusions after randomisation/assignment, together with reasons | This item has been modified for analysis of phase II trials |
| Recruitment | 14a | Dates defining the periods of recruitment and follow-up |  |
|  | 14b | Why the trial ended or was stopped | Not applicable in all trials. |
| Baseline Data | 15 | A table showing baseline demographic and clinical characteristics for each group |  |
| Numbers Analysed | 16* | For each group, number of participants (denominator) included in each analysis and whether the analysis was by original assigned groups (if it is a single arm study, number of participants in single group) | This item has been modified for analysis of phase II trials |
| Outcomes and Estimation | 17a | For each primary and secondary outcome, results for each group, and the estimated effect size and its precision (such as 95% confidence interval) |  |
|  | 17b | For binary outcomes, presentation of both absolute and relative effect sizes is recommended | Not applicable in all trials. |
| Ancillary Analyses | 18 | Results of any other analyses performed, including subgroup analyses and adjusted analyses, distinguishing pre-specified from exploratory |  |
| Harms | 19 | All important harms or unintended effects in each group |  |
| **Discussion** | | | |
| Limitations | 20 | Trial limitations, addressing sources of potential bias, imprecision, and, if relevant, multiplicity of analyses |  |
| Generalisability | 21 | Generalisability (external validity, applicability) of the trial findings |  |
| Interpretation | 22 | Interpretation consistent with results, balancing benefits and harms, and considering other relevant evidence |  |
| **Other Information** | | | |
| Registration | 23 | Registration number and name of trial registry |  |
| Protocol | 24 | Where the full trial protocol can be accessed, if available |  |
| Funding | 25 | Sources of funding and other support (such as supply of drugs), role of funders |  |

*Supplementary Table 6: Modified CONSORT 2010 checklist, with additional guidance to aid analysis of phase II trials*

| **Section/Topic** | **Item Number** | **Item Description** | **Additional Guidance** |
| --- | --- | --- | --- |
| **Title and Abstract** | | | |
|  | 1a | Identification as a randomised trial in the title |  |
|  | 1b | Structured summary of trial design, methods, results, and conclusions | If it has a “structured summary of trial design, methods, results and conclusions”, it can be reported as YES. An in- depth analysis of the abstract is done separately using CONSORT-A. |
| **Introduction** | | | |
| Background and Objectives | 2a | Scientific background and explanation of  rationale |  |
|  | 2b | Specific objectives or hypotheses |  |
| **Methods** | | | |
|  | 3a | Description of trial design (such as parallel, factorial) including allocation ratio |  |
|  | 3b | Important changes to methods after trial commencement (such as eligibility criteria), with reasons |  |
| Participants | 4a | Eligibility criteria for participants collected | Cannot just mention that there are eligibility criteria, must also state what the criteria are. |
|  | 4b | Settings and locations where the data were collected |  |
| Interventions | 5 | The interventions for each group with sufficient details to allow replication, including how and when they were actually administered |  |
| Outcomes | 6a | Completely defined pre-specified primary and secondary outcome measures, including how and when they were assessed |  |
|  | 6b | Any changes to trial outcomes after the trial commenced, with reasons |  |
| Sample Size | 7a | How sample size was determined |  |
|  | 7b | When applicable, explanation of interim analyses and stopping guidelines |  |
| Randomisation: | | | |
| Sequence Generation | 8a | Method used to generate the random  allocation sequence |  |
|  | 8b | Type of randomisation; details of any  restriction (such as blocking and block size) |  |
| Allocation concealment mechanism | 9 | Mechanism used to implement the random allocation sequence (such as sequentially numbered containers), describing any steps taken to conceal the sequence until interventions were assigned |  |
| Implementation | 10 | Who generated the random allocation sequence, who enrolled participants, and who assigned participants to interventions |  |
| Blinding | 11a | If done, who was blinded after assignment to interventions (for example, participants, care providers, those assessing outcomes) and how |  |
|  | 11b | If relevant, description of the similarity of interventions | Not applicable in all trials. |
| Statistical Methods | 12a | Statistical methods used to compare groups for primary and secondary outcomes |  |
|  | 12b | Methods for additional analyses, such as subgroup analyses and adjusted analyses |  |
| **Results** | | | |
| Participant flow (diagram strongly recommended) | 13a | For each group, the numbers of participants who were randomly assigned, received intended treatment, and were analysed for the primary outcome |  |
|  | 13b | For each group, losses and exclusions after randomisation, together with reasons |  |
| Recruitment | 14a | Dates defining the periods of recruitment and follow-up |  |
|  | 14b | Why the trial ended or was stopped | Not applicable in all trials. |
| Baseline Data | 15 | A table showing baseline demographic and clinical characteristics for each group |  |
| Numbers Analysed | 16 | For each group, number of participants (denominator) included in each analysis and whether the analysis was by original assigned groups |  |
| Outcomes and Estimation | 17a | For each primary and secondary outcome, results for each group, and the estimated effect size and its precision (such as 95% confidence interval) |  |
|  | 17b | For binary outcomes, presentation of both absolute and relative effect sizes is recommended | Not applicable in all trials. |
| Ancillary Analyses | 18 | Results of any other analyses performed, including subgroup analyses and adjusted analyses, distinguishing pre-specified from exploratory |  |
| Harms | 19 | All important harms or unintended effects in each group |  |
| **Discussion** | | | |
| Limitations | 20 | Trial limitations, addressing sources of potential bias, imprecision, and, if relevant, multiplicity of analyses |  |
| Generalisability | 21 | Generalisability (external validity, applicability) of the trial findings |  |
| Interpretation | 22 | Interpretation consistent with results, balancing benefits and harms, and considering other relevant evidence |  |
| **Other Information** | | | |
| Registration | 23 | Registration number and name of trial registry |  |
| Protocol | 24 | Where the full trial protocol can be accessed, if available |  |
| Funding | 25 | Sources of funding and other support (such as supply of drugs), role of funders |  |

*Supplementary Table 7: CONSORT 2010 checklist, with additional guidance to aid analysis*

| **Checklist Item** | **Item Number** | **Description** | **Yes (%)** | **No (%)** | **N/A (%)** |
| --- | --- | --- | --- | --- | --- |
| **Administrative Information** | | | |  |  |
| Title | 1 | Descriptive title identifying the study design, population, interventions, and, if applicable, trial acronym | 9 (100) | 0 (0) | 0 (0) |
| Trial Registration | 2a | Trial identifier and registry name. If not yet registered, name of intended registry | 9 (100) | 0 (0) | 0 (0) |
|  | 2b | All items from the World Health Organization Trial Registration Data Set | 8 (89) | 1 (11) | 0 (0) |
| Protocol version | 3 | Date and version identifier | 9 (100) | 0 (0) | 0 (0) |
| Funding | 4 | Sources and types of financial, material, and other support | 8 (89) | 1 (11) | 0 (0) |
| Roles and Responsibilities | 5a | Names, affiliations, and roles of protocol contributors | 9 (100) | 0 (0) | 0 (0) |
|  | 5b | Name and contact information for the trial sponsor | 8 (89) | 1 (11) | 0 (0) |
|  | 5c | Role of study sponsor and funders, if any, in study design; collection, management, analysis, and interpretation of data; writing of the report; and the decision to submit the report for publication, including whether they will have ultimate authority over any of these activities | 7 (78) | 2 (22) | 0 (0) |
|  | 5d | Composition, roles, and responsibilities of the coordinating centre, steering committee, endpoint adjudication committee, data management team, and other individuals or groups overseeing the trial, if applicable (see Item 21a for data monitoring committee) | 6 (67) | 3 (33) | 0 (0) |
| **Introduction** | | | |  |  |
| Background and Rationale | 6a | Description of research question and justification for undertaking the trial, including summary of relevant studies (published and unpublished) examining benefits and harms for each intervention | 8 (89) | 1 (11) | 0 (0) |
|  | 6b | Explanation for choice of comparators | 8 (89) | 1 (11) | 0 (0) |
| Objectives | 7 | Specific objectives or hypotheses | 9 (100) | 0 (0) | 0 (0) |
| Trial Design | 8 | Description of trial design including type of trial (eg, parallel group, crossover, factorial, single group), allocation ratio, and framework (eg, superiority, equivalence, noninferiority, exploratory) | 8 (89) | 1 (11) | 0 (0) |
| **Methods: Participants, interventions, and outcomes** | | | |  |  |
| Study Setting | 9 | Description of study settings (eg, community clinic, academic hospital) and list of countries where data will be collected. Reference to where list of study sites can be obtained | 7 (78) | 2 (22) | 0 (0) |
| Eligibility Criteria | 10 | Inclusion and exclusion criteria for participants. If applicable, eligibility criteria for study centres and individuals who will perform the interventions (eg, surgeons, psychotherapists) | 9 (100) | 0 (0) | 0 (0) |
| Interventions | 11a | Interventions for each group with sufficient detail to allow replication, including how and when they will be administered | 9 (100) | 0 (0) | 0 (0) |
|  | 11b | Criteria for discontinuing or modifying allocated interventions for a given trial participant (eg, drug dose change in response to harms, participant request, or improving/worsening disease) | 6 (67) | 3 (33) | 0 (0) |
|  | 11c | Strategies to improve adherence to intervention protocols, and any procedures for monitoring adherence (eg, drug tablet return, laboratory tests) | 5 (56) | 4 (44) | 0 (0) |
|  | 11d | Relevant concomitant care and interventions that are permitted or prohibited during the trial | 5 (56) | 4 (44) | 0 (0) |
| Outcomes | 12 | Primary, secondary, and other outcomes, including the specific measurement variable (eg, systolic blood pressure), analysis metric (eg, change from baseline, final value, time to event), method of aggregation (eg, median, proportion), and time point for each outcome. Explanation of the clinical relevance of chosen efficacy and harm outcomes is strongly recommended | 8 (89) | 1 (11) | 0 (0) |
| Participant Timeline | 13 | Time schedule of enrolment, interventions (including any run-ins and washouts), assessments, and visits for participants. A schematic diagram is highly recommended (see Figure) | 8 (89) | 1 (11) | 0 (0) |
| Sample Size | 14 | Estimated number of participants needed to achieve study objectives and how it was determined, including clinical and statistical assumptions supporting any sample size calculations | 4 (44) | 5 (56) | 0 (0) |
| Recruitment | 15 | Strategies for achieving adequate participant enrolment to reach target sample size | 5 (56) | 4 (44) | 0 (0) |
| **Methods: Assignment of interventions (for controlled trials)** | | | |  |  |
| Allocation: | | | |  |  |
| Sequence Generation | 16a | Method of generating the allocation sequence (eg, computer-generated random numbers), and list of any factors for stratification. To reduce predictability of a random sequence, details of any planned restriction (eg, blocking) should be provided in a separate document that is unavailable to those who enrol participants or assign interventions | 5 (56) | 4 (44) | 0 (0) |
| Allocation Concealment Mechanism | 16b | Mechanism of implementing the allocation sequence (eg, central telephone; sequentially numbered, opaque, sealed envelopes), describing any steps to conceal the sequence until interventions are assigned | 2 (22) | 7 (78) | 0 (0) |
| Implementation | 16c | Who will generate the allocation sequence, who will enrol participants, and who will assign participants to interventions | 1 (11) | 8 (89) | 0 (0) |
| Blinding (Masking) | 17a | Who will be blinded after assignment to interventions (eg, trial participants, care providers, outcome assessors, data analysts), and how | 4 (44) | 5 (56) | (0) |
|  | 17b | If blinded, circumstances under which unblinding is permissible, and procedure for revealing a participant’s allocated intervention during the trial | 4 (44) | 1 (11) | 4 (44) |
| **Methods: Data collection, management, and analysis** | | | |  |  |
| Data Collection Methods | 18a | Plans for assessment and collection of outcome, baseline, and other trial data, including any related processes to promote data quality (eg, duplicate measurements, training of assessors) and a description of study instruments (eg, questionnaires, laboratory tests) along with their reliability and validity, if known. Reference to where data collection forms can be found, if not in the protocol | 8 (89) | 1 (11) | 0 (0) |
|  | 18b | Plans to promote participant retention and complete follow-up, including list of any outcome data to be collected for participants who discontinue or deviate from intervention protocols | 9 (100) | 0 (0) | 0 (0) |
| Data Management | 19 | Plans for data entry, coding, security, and storage, including any related processes to promote data quality (eg, double data entry; range checks for data values). Reference to where details of data management procedures can be found, if not in the protocol | 8 (89) | 1 (11) | 0 (0) |
| Statistical Methods | 20a | Statistical methods for analysing primary and secondary outcomes. Reference to where other details of the statistical analysis plan can be found, if not in the protocol | 8 (89) | 1 (11) | 0 (0) |
|  | 20b | Methods for any additional analyses (eg, subgroup and adjusted analyses) | 8 (89) | 1 (11) | 0 (0) |
|  | 20c | Definition of analysis population relating to protocol non-adherence (eg, as randomised analysis), and any statistical methods to handle missing data (eg, multiple imputation) | 9 (100) | 0 (0) | 0 (0) |
| **Methods: Monitoring** | | | |  |  |
| Data Monitoring | 21a | Composition of data monitoring committee (DMC); summary of its role and reporting structure; statement of whether it is independent from the sponsor and competing interests; and reference to where further details about its charter can be found, if not in the protocol. Alternatively, an explanation of why a DMC is not needed | 5 (56) | 4 (44) | 0 (0) |
|  | 21b | Description of any interim analyses and stopping guidelines, including who will have access to these interim results and make the final decision to terminate the trial | 7 (78) | 2 (22) | 0 (0) |
| Harms | 22 | Plans for collecting, assessing, reporting, and managing solicited and spontaneously reported adverse events and other unintended effects of trial interventions or trial conduct | 7 (78) | 2 (22) | 0 (0) |
| Auditiing | 23 | Frequency and procedures for auditing trial conduct, if any, and whether the process will be independent from investigators and the sponsor | 6 (67) | 3 (33) | 0 (0) |
| **Ethics and Dissemination** | | | |  |  |
| Research ethics approval | 24 | Plans for seeking research ethics committee/institutional review board (REC/IRB) approval | 4 (44) | 5 (56) | 0 (0) |
| Protocol amendments | 25 | Plans for communicating important protocol modifications (eg, changes to eligibility criteria, outcomes, analyses) to relevant parties (eg, investigators, REC/IRBs, trial participants, trial registries, journals, regulators) | 6 (67) | 3 (33) | 0 (0) |
| Consent or assent | 26a | Who will obtain informed consent or assent from potential trial participants or authorised surrogates, and how (see Item 32) | 7 (78) | 2 (22) | 0 (0) |
|  | 26b | Additional consent provisions for collection and use of participant data and biological specimens in ancillary studies, if applicable | 4 (44) | 5 (56) | 0 (0) |
| Confidentiality | 27 | How personal information about potential and enrolled participants will be collected, shared, and maintained in order to protect confidentiality before, during, and after the trial | 7 (78) | 2 (22) | 0 (0) |
| Declaration of interests | 28 | Financial and other competing interests for principal investigators for the overall trial and each study site | 8 (89) | 1 (11) | 0 (0) |
| Access to data | 29 | Statement of who will have access to the final trial dataset, and disclosure of contractual agreements that limit such access for investigators | 6 (67) | 3 (33) | 0 (0) |
| Ancillary and post-trial care | 30 | Provisions, if any, for ancillary and post-trial care, and for compensation to those who suffer harm from trial participation | 7 (78) | 2 (22) | 0 (0) |
| Dissemination policy | 31a | Plans for investigators and sponsor to communicate trial results to participants, healthcare professionals, the public, and other relevant groups (eg, via publication, reporting in results databases, or other data sharing arrangements), including any publication restrictions | 9 (100) | 0 (0) | 0 (0) |
|  | 31b | Authorship eligibility guidelines and any intended use of professional writers | 7 (78) | 2 (22) | 0 (0) |
|  | 31c | Plans, if any, for granting public access to the full protocol, participant-level dataset, and statistical code | 8 (89) | 1 (11) | 0 (0) |
| **Appendices** | | | |  |  |
| Informed Consent Materials | 32 | Model consent form and other related documentation given to participants and authorised surrogates | 8 (89) | 1 (11) | 0 (0) |
| Biological Specimens | 33 | Plans for collection, laboratory evaluation, and storage of biological specimens for genetic or molecular analysis in the current trial and for future use in ancillary studies, if applicable | 8 (89) | 1 (11) | 0 (100) |

*Supplementary Table 8: Compliance of articles with SPIRIT Checklist*

| **Checklist Item** | **Item Number** | **Description** | **Yes (%)** | **No(%)** | **N/A (%)** |
| --- | --- | --- | --- | --- | --- |
| **Title** | 1 | Identification of the study as a trial | 60 (88) | 8 (12) | 0 (0) |
| **Authors** | 2 | Contact details for the corresponding author | 64 (94) | 4 (6) | 0 (0) |
| **Trial Design** | 3 | Description of the trial design (e.g. parallel, cluster, non-inferiority) | 32 (47) | 36 (53) | 0 (0) |
| **Methods** | | | | | |
| Participants | 4 | Eligibility criteria for participants and the settings where the data were collected | 54 (79) | 14 (21) | 0 (0) |
| Interventions | 5 | Interventions intended for each group | 67 (99) | 1 (1) | 0 (0) |
| Objective | 6 | Specific objective or hypothesis | 64 (94) | 4 (6) | 0 (0) |
| Outcome | 7 | Clearly defined primary outcome for this report | 49 (72) | 19 (28) | 0 (0) |
| Randomisation | 8 | How participants were allocated to interventions | 0 (0) | 4 (6) | 64 (94) |
| Blinding (Masking) | 9 | Whether or not participants, care givers, and those assessing the outcomes were blinded to group assignment | 0 (0) | 1 (1) | 67 (99) |
| **Results** | | | | | |
| Numbers Randomised | 10 | Number of participants allocated to each group | 54 (79) | 14 (21) | 0 (0) |
| Recruitment | 11 | Trial Status | 8 (12) | 60 (88) | 0 (0) |
| Numbers Analysed | 12 | Number of participants analysed in each group | 50 (74) | 18 (26) | 0 (0) |
| Outcome | 13 | For the primary outcome, a result for each group and the estimated effect size and its precision | 54 (79) | 14 (21) | 0 (0) |
| Harms | 14 | Important adverse events or side effects | 47 (69) | 21 (31) | 0 (0) |
| **Conclusions** | 15 | General interpretation of the results | 68 (100) | 0 (0) | 0 (0) |
| **Trial Registration** | 16 | Registration number and name of trial register | 15 (22) | 53 (78) | 0 (0) |
| **Funding** | 17 | Source of funding | 4 (6) | 64 (94) | 0 (0) |

*Supplementary Table 9: Compliance of phase II abstracts of included articles to modified CONSORT-A*

| **Checklist Item** | **Item Number** | **Description** | **Yes (%)** | **No(%)** | **N/A (%)** |
| --- | --- | --- | --- | --- | --- |
| **Title** | 1 | Identification of the study as randomized | 5 (62.5) | 3 (37.5) | 0 (0) |
| **Authors** | 2 | Contact details for the corresponding author | 2 (25) | 0 (0) | 6 (75) |
| **Trial Design** | 3 | Description of the trial design (e.g. parallel, cluster, non-inferiority) | 2 (25) | 6 (75) | 0 (0) |
| **Methods** | | | | | |
| Participants | 4 | Eligibility criteria for participants and the settings where the data were collected | 5 (62.5) | 3 (37.5) | 0 (0) |
| Interventions | 5 | Interventions intended for each group | 8 (100) | 0 (0) | 0 (0) |
| Objective | 6 | Specific objective or hypothesis | 8 (100) | 0 (0) | 0 (0) |
| Outcome | 7 | Clearly defined primary outcome for this report | 7 (87.5) | 1 (12.5) | 0 (0) |
| Randomisation | 8 | How participants were allocated to interventions | 0 (0) | 8 (100) | 0 (0) |
| Blinding (Masking) | 9 | Whether or not participants, care givers, and those assessing the outcomes were blinded to group assignment | 1 (12.5) | 7 (87.5) | 0 (0) |
| **Results** | | | | | |
| Numbers Randomised | 10 | Number of participants randomized to each group | 3 (37.5) | 5 (62.5) | 0 (0) |
| Recruitment | 11 | Trial Status | 0 (0) | 8 (100) | 0 (0) |
| Numbers Analysed | 12 | Number of participants analysed in each group | 1 (12.5) | 7 (87.5) | 0 (0) |
| Outcome | 13 | For the primary outcome, a result for each group and the estimated effect size and its precision | 7 (87.5) | 1 (12.5) | 0 (0) |
| Harms | 14 | Important adverse events or side effects | 1 (12.5) | 7 (87.5) | 0 (0) |
| **Conclusions** | 15 | General interpretation of the results | 8 (100) | 0 (0) | 0 (0) |
| **Trial Registration** | 16 | Registration number and name of trial register | 4 (50) | 4 (50) | 0 (0) |
| **Funding** | 17 | Source of funding | 0 (0) | 8 (100) | 0 (0) |

*Supplementary Table 10: Compliance of abstracts of included articles to CONSORT-A*

| **Section/Topic** | **Item Number** | **Item Description** | **Yes (%)** | **No (%)** | **N/A (%)** |
| --- | --- | --- | --- | --- | --- |
| **Title and Abstract** | | | | | |
|  | 1a | Identification as a randomised trial in the title | 67 (99) | 1 (1) | 0 (0) |
|  | 1b | Structured summary of trial design, methods, results, and conclusions | 55 (81) | 13 (19) | 0 (0) |
| **Introduction** | | | | | |
| Background and Objectives | 2a | Scientific background and explanation of  rationale | 68 (100) | 0 (0) | 0 (0) |
|  | 2b | Specific objectives or hypotheses | 67 (99) | 1 (1) | 0 (0) |
| Methods | | | | | |
|  | 3a | Description of trial design (such as parallel, factorial) including allocation ratio | 56 (82) | 12 (18) | 0 (0) |
|  | 3b | Important changes to methods after trial commencement (such as eligibility criteria), with reasons | 5 (7) | 63 (93) | 0 (0) |
| Participants | 4a | Eligibility criteria for participants collected | 67 (99) | (1) | 0 (0) |
|  | 4b | Settings and locations where the data were collected | 30 (44) | 38 (66) | 0 (0) |
| Interventions | 5 | The interventions for each group with sufficient details to allow replication, including how and when they were actually administered | 68 (100) | 0 (0) | 0 (0) |
| Outcomes | 6a | Completely defined pre-specified primary and secondary outcome measures, including how and when they were assessed | 64 (94) | 4 (6) | 0 (0) |
|  | 6b | Any changes to trial outcomes after the trial commenced, with reasons | 4 (6) | 50 (74) | 14 (20) |
| Sample Size | 7a | How sample size was determined | 24 (35) | 44 (65) | 0 (0) |
|  | 7b | When applicable, explanation of interim analyses and stopping guidelines | 37 (54) | 31 (46) | 0 (0) |
| Sequence Generation | 8a | Method used to generate the random  allocation sequence | 3 (4) | 0 (0) | 65 (96) |
|  | 8b | Type of randomisation; details of any  restriction (such as blocking and block size) | 2 (3) | 3 (4) | 63 (93) |
| Allocation concealment mechanism | 9 | Mechanism used to implement the random allocation sequence (such as sequentially numbered containers), describing any steps taken to conceal the sequence until interventions were assigned | 2 (3) | 3 (4) | 63 (93) |
| Implementation | 10 | Who generated the random allocation sequence, who enrolled participants, and who assigned participants to interventions | 65 (96) | 2 (3) | 1 (1) |
| Blinding | 11a | If done, who was blinded after assignment to interventions (for example, participants, care providers, those assessing outcomes) and how | 2 (3) | 2 (3) | 64 (94) |
|  | 11b | If relevant, description of the similarity of interventions | 1 (1) | 1 (1) | 66 (98) |
| Statistical Methods | 12a | Statistical methods used to compare groups for primary and secondary outcomes | 61 (90) | 7 (10) | 0 (0) |
|  | 12b | Methods for additional analyses, such as subgroup analyses and adjusted analyses | 67 (99) | 1 (1) | 0 (0) |
| **Results** | | | | | |
| Participant flow (diagram strongly recommended) | 13a | For each group, the numbers of participants who were randomly assigned, received intended treatment, and were analysed for the primary outcome | 65 (96) | 3 (4) | 0 (0) |
|  | 13b | For each group, losses and exclusions after randomisation, together with reasons | 51 (75) | 17 (25) | 0 (0) |
| Recruitment | 14a | Dates defining the periods of recruitment and follow-up | 48 (71) | 20 (29) | 0 (0) |
|  | 14b | Why the trial ended or was stopped |  |  |  |
| Baseline Data | 15 | A table showing baseline demographic and clinical characteristics for each group | 59 (87) | 9 (13) | 0 (0) |
| Numbers Analysed | 16 | For each group, number of participants (denominator) included in each analysis and whether the analysis was by original assigned groups | 66 (97) | 2 (3) | 0 (0) |
| Outcomes and Estimation | 17a | For each primary and secondary outcome, results for each group, and the estimated effect size and its precision (such as 95% confidence interval) | 65 (96) | 3 (4) | 0 (0) |
|  | 17b | For binary outcomes, presentation of both absolute and relative effect sizes is recommended | 0 (0) | 0 (0) | 68 (100) |
| Ancillary Analyses | 18 | Results of any other analyses performed, including subgroup analyses and adjusted analyses, distinguishing pre-specified from exploratory | 67 (99) | 1 (1) | 0 (0) |
| Harms | 19 | All important harms or unintended effects in each group | 63 (93) | 5 (7) | 0 (0) |
| **Discussion** | | | | | |
| Limitations | 20 | Trial limitations, addressing sources of potential bias, imprecision, and, if relevant, multiplicity of analyses | 37 (54) | 31 (46) | 0 (0) |
| Generalisability | 21 | Generalisability (external validity, applicability) of the trial findings | 67 (99) | 1 (1) | 0 (0) |
| Interpretation | 22 | Interpretation consistent with results, balancing benefits and harms, and considering other relevant evidence | 68 (100) | 0 (0) | 0 (0) |
| **Other Information** | | | | | |
| Registration | 23 | Registration number and name of trial registry | 40 (59) | 28 (41) | 0 (0) |
| Protocol | 24 | Where the full trial protocol can be accessed, if available | 53 (78) | 15 (12) | 0 (0) |
| Funding | 25 | Sources of funding and other support (such as supply of drugs), role of funders | 51 (75) | 17 (25) | 0 (0) |

*Supplementary Appendix Table 11: Compliance of articles to CONSORT 2010 Statement, modified for analysis of phase II trials*

| **Section/Topic** | **Item Number** | **Item Description** | **Yes (%)** | **No (%)** | **N/A (%)** |
| --- | --- | --- | --- | --- | --- |
| **Title and Abstract** | | | | | |
|  | 1a | Identification as a randomised trial in the title | 5 (62.5) | 3 (37.5) | 0 (0) |
|  | 1b | Structured summary of trial design, methods, results, and conclusions | 8 (100) | 0 (0) | 0 (0) |
| **Introduction** | | | | | |
| Background and Objectives | 2a | Scientific background and explanation of  rationale | 8 (100) | 0 (0) | 0 (0) |
|  | 2b | Specific objectives or hypotheses | 8 (100) | 0 (0) | 0 (0) |
| Methods | | | | | |
|  | 3a | Description of trial design (such as parallel, factorial) including allocation ratio | 4 (50) | 4 (50) | 0 (0) |
|  | 3b | Important changes to methods after trial commencement (such as eligibility criteria), with reasons | 2 (25) | 0 (0) | 6 (75) |
| Participants | 4a | Eligibility criteria for participants collected | 7 (87.5) | 1 (12.5) | 0 (0) |
|  | 4b | Settings and locations where the data were collected | 3 (37.5) | 5 (62.5) | 0 (0) |
| Interventions | 5 | The interventions for each group with sufficient details to allow replication, including how and when they were actually administered | 3 (37.5) | 5 (62.5) | 0 (0) |
| Outcomes | 6a | Completely defined pre-specified primary and secondary outcome measures, including how and when they were assessed | 8 (100) | 0 (0) | 0 (0) |
|  | 6b | Any changes to trial outcomes after the trial commenced, with reasons | 2 (25) | 0 (0) | 6 (75) |
| Sample Size | 7a | How sample size was determined | 3 (37.5) | 5 (62.5) | 0 (0) |
|  | 7b | When applicable, explanation of interim analyses and stopping guidelines | 1 (12.5) | 0 (0) | 7 (87.5) |
| Sequence Generation | 8a | Method used to generate the random  allocation sequence | 3 (37.5) | 5 (62.5) | 0 (0) |
|  | 8b | Type of randomisation; details of any  restriction (such as blocking and block size) | 6 (75) | 2 (25) | 0 (0) |
| Allocation concealment mechanism | 9 | Mechanism used to implement the random allocation sequence (such as sequentially numbered containers), describing any steps taken to conceal the sequence until interventions were assigned | 3 (37.5) | 5 (62.5) | 0 (0) |
| Implementation | 10 | Who generated the random allocation sequence, who enrolled participants, and who assigned participants to interventions | 1 (12.5) | 7 (87.5) | 0 (0) |
| Blinding | 11a | If done, who was blinded after assignment to interventions (for example, participants, care providers, those assessing outcomes) and how | 1 (12.5) | 7 (87.5) | 0 (0) |
|  | 11b | If relevant, description of the similarity of interventions | 1 (12.5) | 5 (62.5) | 2 (25) |
| Statistical Methods | 12a | Statistical methods used to compare groups for primary and secondary outcomes | 8 (100) | 0 (0) | 0 (0) |
|  | 12b | Methods for additional analyses, such as subgroup analyses and adjusted analyses | 2 (25) | 3 (37.5) | 3 (37.5) |
| **Results** | | | | | |
| Participant flow (diagram strongly recommended) | 13a | For each group, the numbers of participants who were randomly assigned, received intended treatment, and were analysed for the primary outcome | 6 (75) | 2 (25) | 0 (0) |
|  | 13b | For each group, losses and exclusions after randomisation, together with reasons | 7 (87.5) | 1 (12.5) | 0 (0) |
| Recruitment | 14a | Dates defining the periods of recruitment and follow-up | 4 (50) | 4 (50) | 0 (0) |
|  | 14b | Why the trial ended or was stopped | 0 (0) | 0 (0) | 8 (100) |
| Baseline Data | 15 | A table showing baseline demographic and clinical characteristics for each group | 7 (87.5) | 1 (12.5) | 0 (0) |
| Numbers Analysed | 16 | For each group, number of participants (denominator) included in each analysis and whether the analysis was by original assigned groups | 7 (87.5) | 1 (12.5) | 0 (0) |
| Outcomes and Estimation | 17a | For each primary and secondary outcome, results for each group, and the estimated effect size and its precision (such as 95% confidence interval) | 8 (100) | 0 (0) | 0 (0) |
|  | 17b | For binary outcomes, presentation of both absolute and relative effect sizes is recommended | 0 (0) | 0 (0) | 8 (100) |
| Ancillary Analyses | 18 | Results of any other analyses performed, including subgroup analyses and adjusted analyses, distinguishing pre-specified from exploratory | 5 (62.5) | 0 (0) | 3 (37.5) |
| Harms | 19 | All important harms or unintended effects in each group | 6 (75) | 2 (25) | 0 (0) |
| **Discussion** | | | | | |
| Limitations | 20 | Trial limitations, addressing sources of potential bias, imprecision, and, if relevant, multiplicity of analyses | 3 (37.5) | 5 (62.5) | 0 (0) |
| Generalisability | 21 | Generalisability (external validity, applicability) of the trial findings | 8 (100) | 0 (0) | 0 (0) |
| Interpretation | 22 | Interpretation consistent with results, balancing benefits and harms, and considering other relevant evidence | 5 (62.5) | 0 (0) | 3 (37.5) |
| **Other Information** | | | | | |
| Registration | 23 | Registration number and name of trial registry | 6 (75) | 2 (25) | 0 (0) |
| Protocol | 24 | Where the full trial protocol can be accessed, if available | 5 (62.5) | 0 (0) | 3 (37.5) |
| Funding | 25 | Sources of funding and other support (such as supply of drugs), role of funders | 6 (75) | 2 (25) | 0 (0) |

*Supplementary Appendix Table 12: Compliance of phase III articles to CONSORT 2010 Statement*
